# How high can the fatigue strength of metals be achieved?

**DOI:** 10.1093/nsr/nwaf332

**Published:** 2025-08-15

**Authors:** Zikuan Xu, Xiaolin Su, Peng Zhang, Bin Wang, Zhan Qu, Aiping Wang, Zhefeng Zhang

**Affiliations:** Materials Fatigue and Fracture Division, Shenyang National Laboratory for Materials Science, Institute of Metal Research, Chinese Academy of Sciences, China; Materials Fatigue and Fracture Division, Shenyang National Laboratory for Materials Science, Institute of Metal Research, Chinese Academy of Sciences, China; School of Materials Science and Engineering, University of Science and Technology of China, China; Materials Fatigue and Fracture Division, Shenyang National Laboratory for Materials Science, Institute of Metal Research, Chinese Academy of Sciences, China; School of Materials Science and Engineering, University of Science and Technology of China, China; Materials Fatigue and Fracture Division, Shenyang National Laboratory for Materials Science, Institute of Metal Research, Chinese Academy of Sciences, China; Materials Fatigue and Fracture Division, Shenyang National Laboratory for Materials Science, Institute of Metal Research, Chinese Academy of Sciences, China; Research and Development Centre, Zenith Steel Group (Huai'an) New Material Company Limited, China; Materials Fatigue and Fracture Division, Shenyang National Laboratory for Materials Science, Institute of Metal Research, Chinese Academy of Sciences, China; School of Materials Science and Engineering, University of Science and Technology of China, China

## Abstract

This study presents four principles for enhancing metallic fatigue resistance, achieving record-high fatigue strength in cold-drawn pearlitic steel with oriented nano-scale lamellar microstructure and extremely small inclusions.

Metals are ubiquitous in structural applications due to their exceptional balance of strength and ductility. However, in applications involving cyclic loading, fatigue resistance becomes the paramount concern, because materials can fail catastrophically after a specified number of cycles, even at stresses significantly below their yield or tensile strength [1]. Consequently, fatigue strength (FS) is defined as the maximum stress a material can endure without failure after a certain number of cycles, typically 10^7^ in high-cycle fatigue (HCF) [1]. This property is crucial for structural components, whose durability and reliability are indispensable. In addition, an increase in FS can also enable components to withstand the same load without failure at smaller cross-sectional areas, thereby achieving lightweight components which can improve energy efficiency and also reduce carbon emissions to address climate change.

The nucleation of microcracks in ductile metals is a consequence of the to-and-fro motion of dislocations during cyclic loading [1]. Therefore, improving FS of metallic materials mainly involves two distinct strategies: (i) preventing fatigue damage. In defect-free metals, optimal FS could be achieved by completely suppressing dislocation motion; whereas in metals containing defects, the matrix near the defects needs to exhibit a strain-hardening capability in order to effectively prevent the continuous movement of dislocations. (ii) Prolonging the fatigue damage process. This necessitates detailed characterization of fatigue

damage evolution, such as studying the dynamic changes in dislocation configurations and their governing mechanisms. Here, we propose four principles for anti-fatigue design of metallic materials using the first strategy.

## ONE OF THESE PRINCIPLES REGARDS ACHIEVING HIGH ELASTIC MODULUS TO PROVIDE HIGH THEORETICAL FS

As the metallic material with the highest Young's modulus among commonly used metals (at room temperature: steels: ∼210 GPa; aluminum alloys: ∼70 GPa; copper alloys: ∼120 GPa; titanium alloys: ∼110 GPa and magnesium alloys: ∼ 45 GPa), steel has the highest recorded strength up to ∼7 GPa [2]. Considering that a perfectly smooth sample containing no inclusions, holes or cracks should be immune to fatigue cracking when the applied stress is lower than its elastic limit, the steel with the highest modulus and consequently highest strength should have the highest theoretical FS. Therefore, to achieve the highest FS to date, steel must be first choice among the various metallic materials.

## ANOTHER PRINCIPLE IS TO MAKE FINE, UNIFORM AND STABLE MICROSTRUCTURE

**Figure 1.**
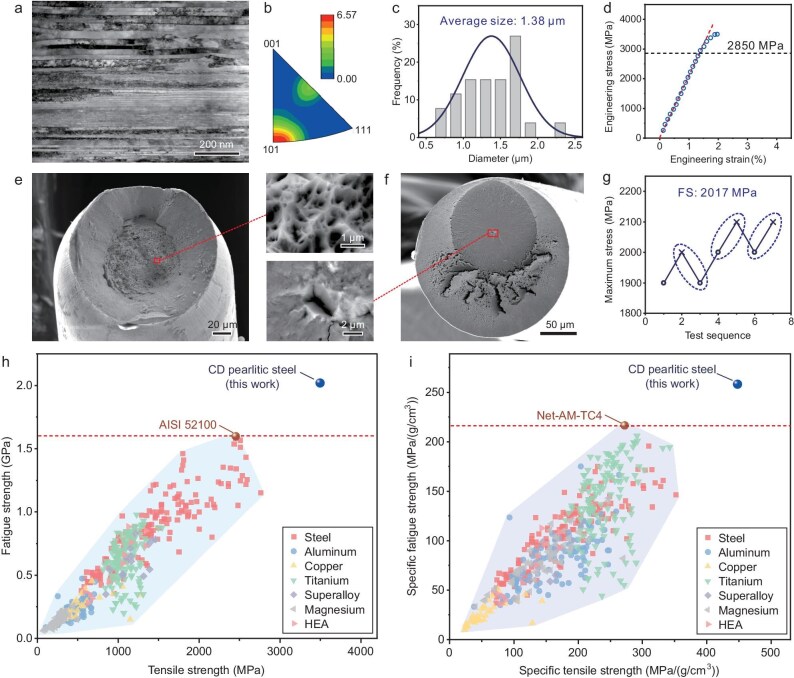
Microstructure and properties of cold-drawn pearlitic steel wire conforming to our proposed principles. Fine, uniform and stable microstructure: (a) TEM image of the longitudinal section; (b) inverse pole figure of the longitudinal section showing <110> and <112> texture existing. Smallest possible inclusion or defect size: (c) histogram and the fitted normal distribution curve of inclusion diameter. Optimal tensile properties: (d) engineering stress vs strain curve; (e) fracture of tensile sample showing the nano-scale dimples. (f) SEM image showing the fatigue cracking mode. (g) Fatigue strength staircase diagram. (h) Fatigue strength at *R* = 0.1 vs tensile strength for our work and for metals reported in the literature (Tables S2–S10). (i) Specific fatigue strength at *R* = 0.1 vs specific tensile strength for our work and the metals reported in the literature available (Tables S2–S10). HEA: high-entropy alloys. AM: additively manufactured.

Metallic materials with finer microstructure generally have better fatigue resistance, because the energy accumulated

by the piling-up of dislocations would be less in smaller grains under the same load [3]. Fortunately, pearlite steel exhibits a nano-scale lamellar, uniform and stable microstructure. Additionally, the cold-drawn (CD) process is adopted here which can eliminate the 45° oriented pearlite layer and form columnar grains with textures (<110> and <112>) in the drawing direction (Fig. 1a, b), making the strength difference in the entire material even smaller. After the CD process, a microstructure with a minimum interlamellar spacing of ∼20 nm was obtained (Fig. S1). The plastic deformation mechanisms of such nano-scale laminated microstructures were revealed by large-scale molecular dynamics simulations [4].

## THE THIRD PRINCIPLE RELATES TO MINIMIZING THE SIZE OF INCLUSIONS OR DEFECTS

Large-scale inclusions or defects within the metallic materials can exacerbate fatigue failure by generating significant stress concentrations in their vicinity [5]. In contrast, material containing smaller inclusions or defects should exhibit a higher FS that is closer to its theoretical FS, which demonstrates a strong correlation with its elastic limit as mentioned above. The inclusions in our CD pearlite steel are extremely small (average size 1.38 μm, Fig. 1c). In addition, optimizing the morphology, reducing the modulus difference between inclusions and matrix, or forming a coherent interface between inclusions and matrix [6] should also reduce stress concentrations.

## THE FOURTH PRINCIPLE IS TO ACHIEVE OPTIMAL TENSILE PROPERTIES INCLUDING ELASTIC LIMIT AND WORK-HARDENING ABILITY

The uniaxial tensile properties of our CD pearlitic steel were evaluated (Fig. 1d). The steel exhibited an ultimate tensile strength of 3525 MPa and an elastic limit of ∼2850 MPa, achieved through the application of three strengthening mechanisms: solid solution hardening, work hardening (Figs S2, S3) and grain boundary strengthening. Tensile fracture of our CD pearlitic steel has an ∼33% reduction in cross-sectional area (Fig. 1e); furthermore, nano-scale dimples are found on its tensile fracture surfaces, which indicates that it still has a certain degree of ductility (Fig. 1e). Obviously, we did not choose the steel wire with the highest strength of ∼7 GPa because it would make the steel wire lose its work-hardening ability [2]. Considering that inclusion-free steel is a vision of perfection that is currently unachievable, the work-hardening ability is necessary to accommodate the stress concentration caused by inclusions [7,8].

Subsequently, the FS of the CD pearlitic steel was tested. In general, most fatigue tests of steel wires are performed using a rotational bending machine, as specified in the ASTM E2948-22 standard. However, as mentioned by the standard, under rotational bending, the stress is graduated from the sample surface to the center, with the stress decreasing from maximum to zero. Consequently, fatigue cracks tend to initiate exclusively near the sample surface, resulting in a higher FS compared to that observed in uniaxial fatigue tests [1]. Accordingly, we designed a special fixing device (Fig. S4) that achieved non-destructive fixation of steel wires through simultaneously bonding, wrapping and clamping. Using this device, we successfully detected the FS of the steel wires (Fig. 1f, g) under uniaxial tension-tension loading for the first time with a stress ratio *R* of 0.1 (where *R* is the ratio of the minimum stress to the maximum stress in the cycle).

As expected, the FS (*R* = 0.1) of our CD pearlitic steel is as high as 2017 MPa, which is the highest FS among all the materials to date (Fig. 1h). It can be seen that the fatigue crack initiated around the interface between inclusion and matrix but not originated from the sample surface (Fig. 1f). This indicates that the stress concentration in the surrounding microstructure of the inclusion exceeds its elastic limit and the dislocations begin to accumulate, leading to fatigue damage and cracking. The fracture surfaces exhibit classic fatigue fracture morphologies [9]: a relatively smooth, circular ‘mirror’ region emanating from the cracking source (Fig. S5) and a rougher surface characteristic of branching cracks beyond the mirror zone (Fig. 1f).

In general, three failure mechanisms compete in pearlitic steel fatigue: surface slip band cracking, pearlitic lamellar interface cracking and debonding at the particle/matrix interface. Inclusion/matrix debonding dominates as the critical failure mode. Weak interfacial bonding and stress concentration at large inclusions create localized weakness, resulting in the FS being lower than the elastic limit (2850 MPa). Surface slip band cracking of the matrix is effectively suppressed due to exceptional material homogeneity and ultra-high elastic limit, making surface initiation less probable than subsurface inclusion failure (Fig. S6). Pearlitic lamellar interface cracking is inherently prevented as the lamellae are aligned with the loading direction (Schmid factor equals zero), eliminating resolved shear stress in the lamellar structure.

For comprehensive evaluation of the mechanical properties of our CD pearlitic steel, the results are further compared with other metals from the perspectives of tensile strength and FS (Fig. 1h). The advantage of our CD pearlitic steel is further underscored by its FS being at least 25% higher than the highest FS record of 1600 MPa in our recent study [10]. To further highlight the high fatigue resistance of the CD pearlitic steel, the specific strength and specific FS of those metals are summarized (Fig. 1i). It is very encouraging to find that the steel has once again become the material with the highest specific FS (258 MPa/(g/cm^3^)) to date among all kinds of metallic materials, including steels, aluminum alloys, titanium alloys, magnesium alloys, copper alloys, superalloys and high-entropy alloys. Additionally, the low price of steel further enhances its potential advantages in manufacturing fatigue-resistant components.

In summary, we propose four principles for metallic materials to achieve excellent fatigue resistance, including: (1) high elastic modulus; (2) fine, uniform and stable microstructure; (3) the smallest possible inclusion or defect size; and (4) optimal tensile properties. The metals with high elastic modulus and fine, uniform and stable microstructure would have high theoretical FS. The inclusion or defect with the smallest possible size and the corresponding optimal tensile properties, including elastic limit and work-hardening ability, would allow the actual FS to be close to its theoretical value. Guided by these principles, the highest FS to date is achieved through a pearlitic steel with a simple cold-drawing process, which is compatible with existing industry processes. This encouraging finding demonstrates that the current four principles are beneficial for the anti-fatigue design of metallic materials. These principles could be extended and tailored to fit many other metals, especially the principle of achieving optimal tensile properties, which may be applicable to the heat treatment of many metallic materials which can then be used in a wide range of future applications in the vehicle, aerospace and machinery industries.

## Supplementary Material

nwaf332_Supplementary_data
