# Supplementary material for: How high can the fatigue strength of metals be achieved?
Source: Natl Sci Rev. 2025 Aug 15;12(9):nwaf332. doi: 10.1093/nsr/nwaf332 (PMC12421570; doi:10.1093/nsr/nwaf332)
Supplement: nwaf332_Supplementary_data [file nwaf332_supplementary_data.pdf]

Supplementary Material

How high can the fatigue strength of metals be achieved?

Zikuan Xu,[1,]† Xiaolin Su,[1,2,]† Peng Zhang,[1,2,]* Bin Wang,[1] Zhan Qu,[1] Aiping Wang,[3] Zhefeng Zhang[1,2,]*

[1]Shenyang National Laboratory for Materials Science, Institute of Metal Research, Chinese Academy of Sciences, Shenyang 110016, China;

[2]School of Materials Science and Engineering, University of Science and Technology of China, Shenyang 110016, China;

[3]Research and Development Centre, Zenith Steel Group (Huai'an) New Material Company Limited, Huai'an 223001, China.

†These authors contributed equally to this work.

*Corresponding authors. E-mails: pengzhang@imr.ac.cn; zhfzhang@imr.ac.cn

Note 1: Materials

Undeformed pearlite steel consisted of alternating layers of ferrite and cementite was selected in this investigation. The steel wire was produced by cold drawing (CD) process at Zenith Steel Group (Huai'an) New Material Co., Ltd., China. Wire rods (diameter 5.5 mm and chemical composition 0.86 wt% C, 0.55 wt% Mn, 0.18 wt% Si and balance Fe) were used for CD processes. The steel wire rods were first cold-drawn to a diameter of 1.53 mm. Then, the steel wires were heated at ~ 1300 K for austenization followed by a pearlite transformation. Subsequently, the steel wires were brass plated and continued to be cold-drawn to a diameter of 225 μm through 26 passes at a speed of 52 m/min. Eventually, tensile and HCF samples with a gauge length of 140 mm and 30 mm were tailored.

Note 2: Mechanical tests

Tensile tests were performed on an INSTRON 8871 testing machine at a strain rate of 2×10^{-4} s^{-1} at room temperature in air. The engineering tensile strain was determined by $\varepsilon = \Delta l/l_0$, where

l_0 and Δl are the initial gauge length and the length change of the wires, respectively. Length changes due to the initial adjustment between wires and deformation of the machine were subtracted from the total measured length changes. The engineering tensile stress was determined by $\sigma = F/S_0$, where F is the force and S_0 is the initial cross-sectional area of the wire. The elastic limit was defined as the stress at a residual elongation of 0.01%. Ten tensile tests were conducted to ensure the repeatability and credibility of the results. HCF tests, with a load ratio (ratio of minimum to maximum stress) of $R = 0.1$, were conducted on a SHIMADZU MMT-101N fatigue tester at a frequency of 40 Hz using our special fixing device. Fatigue tests were stopped when the specimen failed or achieved a lifetime of 10^7 cycles.

Note 3: Microstructure and fracture examination

Scanning electron microscopy (SEM) observations were performed by a ZEISS Sigma 500 field-emission scanning electron microscope. Samples were ground from 400 grit to 1500 grit emery papers and then etched with a mixture of perchloric acid and alcohol with a volume ratio of 1:9. The polishing voltage was 12 V and the polishing time was 24 seconds. Microstructures were characterized by 1) transmission Kikuchi diffraction (TKD) technology using the ZEISS Sigma 500 SEM with an operating voltage of 30 kV and step size 10 nm and 2) transmission electron microscopy (TEM) with an FEI Tecnai F30 microscope operated at 300 kV. TKD data were analysed with Aztec Crystal software. Fatigue fracture morphologies following HCF tests were observed using the ZEISS Sigma 500 SEM operating at 20 kV.

Note 3: Fatigue Test Setup

Directly clamping or wrapping the steel wire will bring additional stress concentration and wear problems during the fatigue test. Therefore, we have designed a set of sample preparation and mounting methods to fix the steel wire through simultaneously bonding, wrapping and clamping.

Preparation

The maximum load of the steel wire in the experiments is between 75 N and 84 N, and the stress ratio is 0.1. The bonding force only needs to be able to share half of the total force, which is ~ 40 N. The bonding shear strength of the epoxy used in this study is generally 10 MPa, while the cyclic bonding shear strength (σ_{CBS}) is not known to us and was set to 1 MPa for safety. Then, the required bonding length can be calculated to be 56 mm:

$$L_{\text{bonding}} = \frac{F_{\text{bonding}}}{\pi d \sigma_{\text{CBS}}}, \tag{S1}$$

where d is the diameter of the sample. By looking around, we found some disposable droppers with the length of about 80 mm, which is very suitable as a container for epoxy.

Use a piece of wood as the pallet, paste tape and add cardboard on the pallet for quick cleaning when the epoxy overflows. Use a trimmed disposable dropper as the container and an aluminium ring as the lock to increase fixing force through friction. An injection hole needs to be cut out on the container in advance (Fig. S1a).

Fatigue sample production

Firstly, fix one end of the wire with a butterfly bolt, sequentially thread a container, three locks and a plug onto the wire, then thread a plug, three locks and a container onto the wire in sequence. The direction of plugs and containers needs to be ensured to be oriented correctly.

Secondly, apply tension to the wire and fix the other end of the wire with a butterfly bolt. The gauge section (which is 30 mm here) of the wire should be set. Besides, 20 cm of wire at both ends should be left for fixing by wrapping and gripping in the next step. The above purpose can be achieved by adjusting the position of plugs, locks, containers and wire holders (Fig. S1a). Squeeze the aluminium ring with pliers to deform and fix it on the steel wire. Fix the container onto the pallet and plug it securely.

Finally, inject the epoxy into the container through the injection hole. After the epoxy solidifies, the sample is ready (Fig. S1a). In this example, three fatigue samples were prepared at once.

Fatigue sample mounting

The required device as a whole is shown in Fig. S1b. The items used include a screw with a length of 150 mm, a spring with an elasticity coefficient of 5 N/mm, and three different types of fixtures for different purposes. Besides, due to the deviation of the sample from the loading centre, four lateral holders are required to ensure that resonance does not occur. Fixture 1 bears the bonding force of the epoxy and the container. Fixture 2 ensures that the container is not bounced back by the spring when the load reaches its minimum. Fixture 3 connects our device with the fatigue machine and can be installed beforehand. The remaining steps are as follows.

Firstly, assemble the bolts, springs and fixture 1 with nuts. The downward pressure of the spring needs to be adjusted according to the load. Here, the combined force of the two spring preloads is set to be 40 N as mentioned before. By limiting with double nuts, the spring preloads are retained and would not release during the cyclic loading process.

Secondly, fix the two ends of the wire by wrapping and clamping, and load it to the average force of the fatigue load.

Thirdly, mount the assembled device onto the sample and connect it to the fixture 3, securing it with double nuts. Afterwards, load it to the maximum value of fatigue load.

Fourthly, put fixture 2 onto the sample and securing it with nuts. Ensure that fixture 2 solidly holds on to the container.

Fifthly, measure the distance between the sample and the column of fatigue tester, install the lateral holder, and ensure that the distance remains unchanged after the installation.

Finally, unload it carefully until the wire slightly bends, reset the load, and then begin fatigue testing.

Final result

The load of fatigue testing is very stable (Fig. S1c). The samples break in the gauge section after the fatigue test (Fig. S1d).

Note 4: Microstructure

We performed TKD analysis to characterize dislocation density before the fatigue test. The average dislocation density in the material obtained by the TKD is 2 × 10^{16}/m^2. However, as shown in Fig. S2, the GND density distribution varies significantly across different regions. Even only in one region, the GND density ranges from 1 × 10^{16}/m^2 to 3 × 10^{16}/m^2 (Fig. S3). Due to the strain introduced in the drawing process, the solution rate of TKD is less than 70%. The TEM images before and after fatigue reveal the dislocation configuration, as shown in Fig. S6. As can be seen, there is virtually no change.

For the microstructure of the matrix, no dislocation cells were observed. This aligns with literature reports stating that dislocation cells are rarely observed when lamellar spacing (λ) is below ~200 nm [1].

Note 5: Statistical analysis of fatigue limit

We conducted a detailed statistical analysis on the fatigue strength based on the Standard ISO 12107:2012: *Metallic materials - Fatigue testing - Statistical planning and analysis of data*, which is elaborated below.

The data of the fatigue limit corresponding to 10^7 cycles are generated by sequentially conducting fatigue tests and using the method known as the staircase method, as shown in Fig. 2a in the main text. Three pairs of data points of failure and non-failure were obtained by alternating the stress levels with a stress step σ_d = 100 MPa, where σ_d is far less than 5% of the fatigue strength. The average value of the fatigue strength can be estimated by arranging the stress levels in ascending order and counting the frequencies of failure and non-failure of the specimens tested at different stress levels:

$$\mu_y = \sigma_0 + \sigma_d \left(\frac{A}{C} \pm \frac{1}{2} \right), \tag{S2}$$

where σ_0 is the stress of the first run-out sample. σ_0 would be 1900 MPa as shown in Fig. 2a in the main text. Other parameters are calculated by:

$$A=\sum_{i=1}^{l} if_i;\ B=\sum_{i=1}^{l} i^2 f_i;\ C=\sum_{i=1}^{l} f_i;\ D=\frac{BC-A^2}{C^2}, \tag{S3}$$

where l is the number of stress levels, f_i is the number of events. In Eq. (S3), taking the value of $\pm$ 1/2 equal to −1/2 when the event analysed is a failure, and +1/2 when the event analysed is a non-failure. The variance for evaluating the statistical distribution of the fatigue limit is written as:

$$\begin{aligned} \sigma_y &= 1.62\sigma_\mathrm{d}\left(D+0.029\right) \text{ for } \left(D>0.3\right) \\ \sigma_y &= 0.53\sigma_\mathrm{d} \text{ for } \left(D\le 0.3\right) \end{aligned}, \tag{S4}$$

By applying the failure events of the experimental results in Fig. 2a in the main text,

$$A=5;\ B=9;\ C=3;\ D=0.22, \tag{S5}$$

where the analysis process is shown in Table S1. Therefore,

$$\begin{aligned} \mu_y &= 1900+100\times\left(\frac{5}{3}-\frac{1}{2}\right)=2017\ \text{MPa} \\ \sigma_y &= 0.53\sigma_\mathrm{d}=53\ \text{MPa} \end{aligned}. \tag{S6}$$

Note 6: Fatigue strength evaluation

Table S2-S10 displays the data of tensile strength and fatigue strength of various metallic materials, including steels, aluminium alloys, titanium alloys, magnesium alloys, copper alloys, superalloys and high-entropy alloys. The densities used to calculate the specific tensile strength and specific fatigue strength are 7.85 g/cm^3 (steels, high-entropy alloys), 2.70 g/cm^3 (aluminium alloys), 8.92 g/cm^3 (copper alloys), 4.51 g/cm^3 (titanium alloys), 8.40 g/cm^3 (superalloys) and 1.80 g/cm^3 (magnesium alloys).

The fatigue strength data obtained at different stress ratios were reconciled by the well-known Goodman equation [2]:

$$\frac{\sigma_{aR}}{\sigma_{a-1}} + \frac{\sigma_{mR}}{\sigma_{a-1}} = 1, \quad \text{(S7)}$$

where σ_{aR} and σ_{a-1} are respectively the stress amplitudes at stress ratios of *R* and -1, σ_{mR} is the mean stress at a stress ratio of *R*, σ_{UTS} is the ultimate tensile strength. Additionally, σ_{fR} and $\sigma_{f0.1}$ are the maximum stress at stress ratios of *R* and 0.1, respectively. With Eq. (S7), the fatigue strength data tested at $R = 0.1$ and at different *R* values can be mutually convertible.

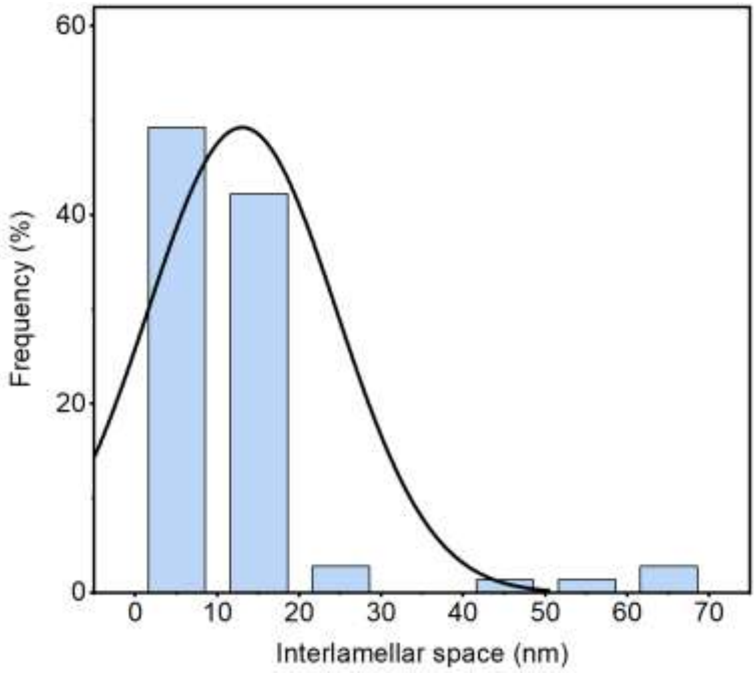

Figure S1. Interlamellar spacing distribution. The average interlamellar space of the as-drawn wire is less than 20 nm.

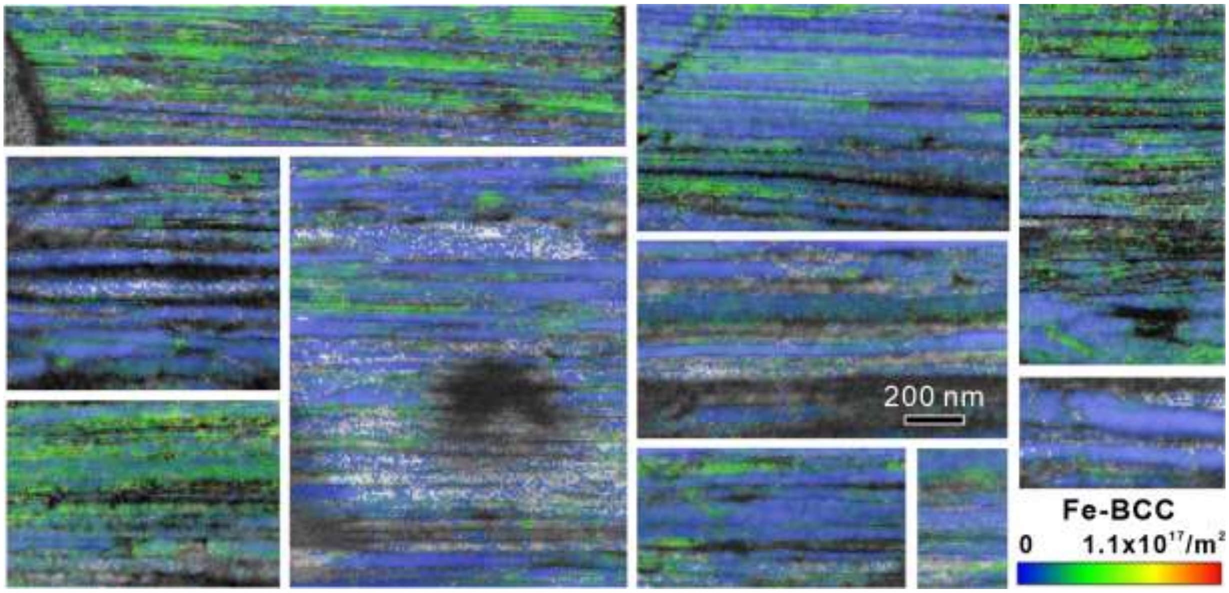

Figure S2. GND of the CD pearlitic steel as drawn. The average dislocation density in the material obtained by the TKD is $2 \times 10^{16}/m^2$.

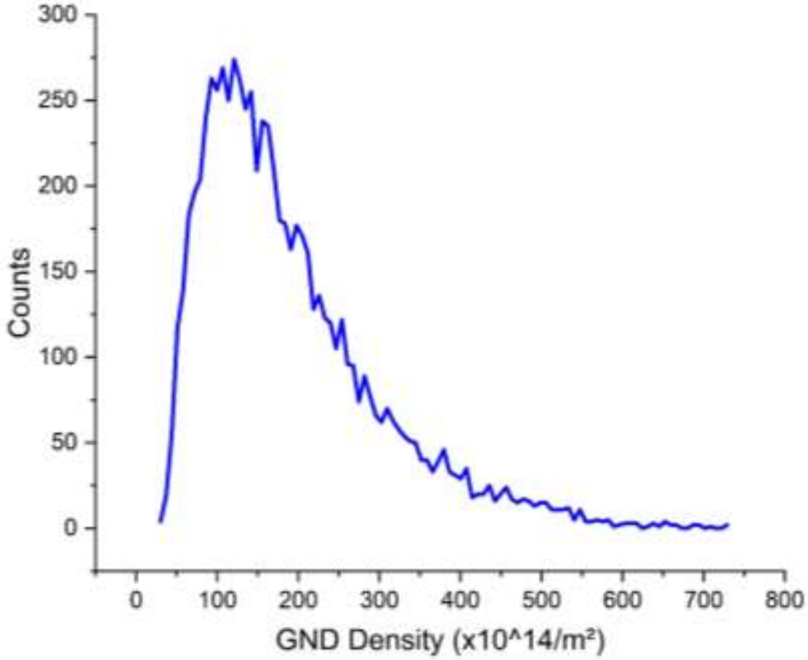

Figure S3. GND distribution of the as-drawn pearlitic wire in one region.

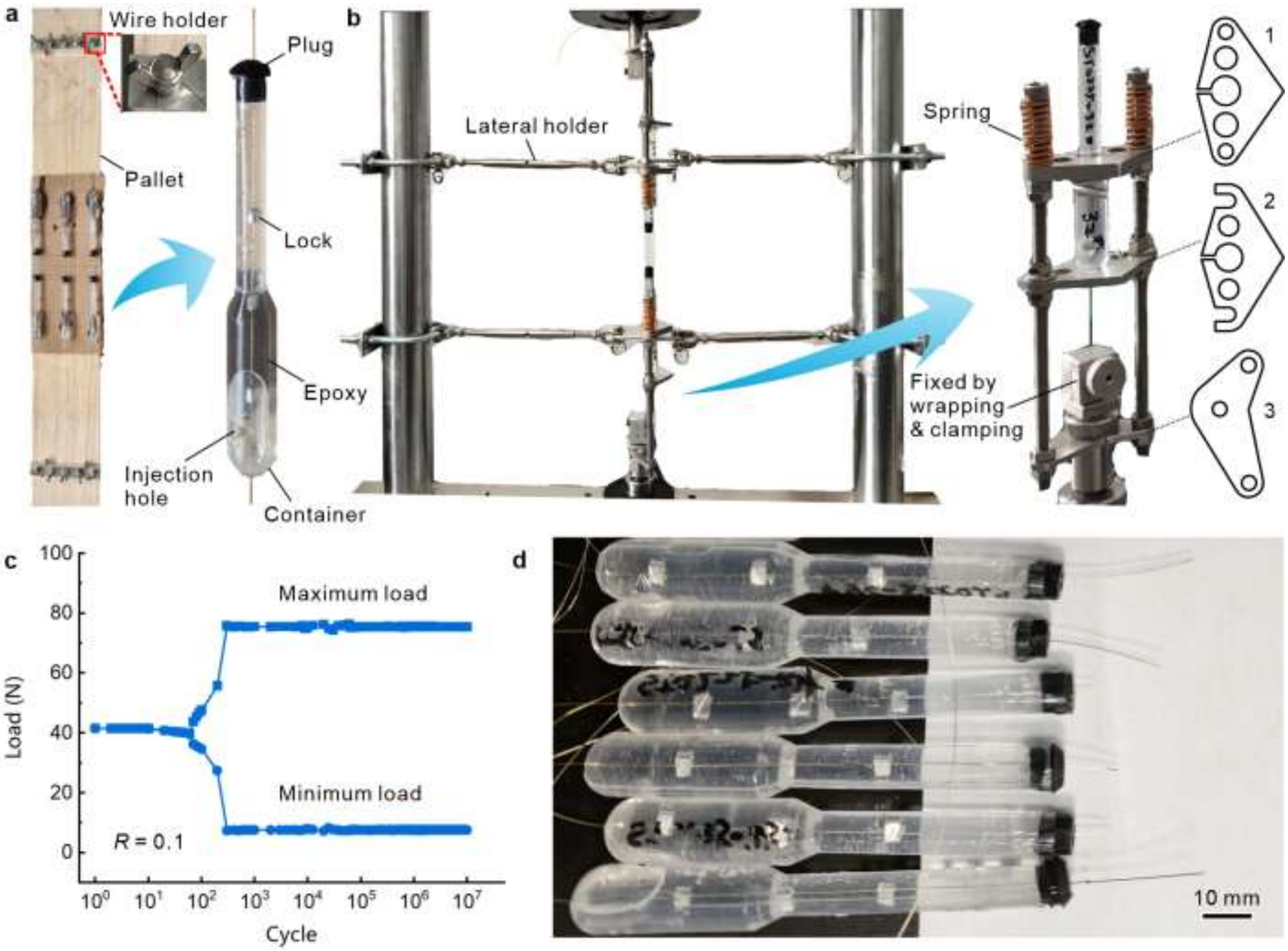

Figure S4. Our fatigue test setup. (a) Preparation of fatigue samples. (b) Alignment, wrapping and clamping system. (c) Recorded load values during the fatigue test. (d) Wire samples after the fatigue test. The stable load during the testing process, as well as the samples that were broken in the working section, ensured the validity of the testing data.

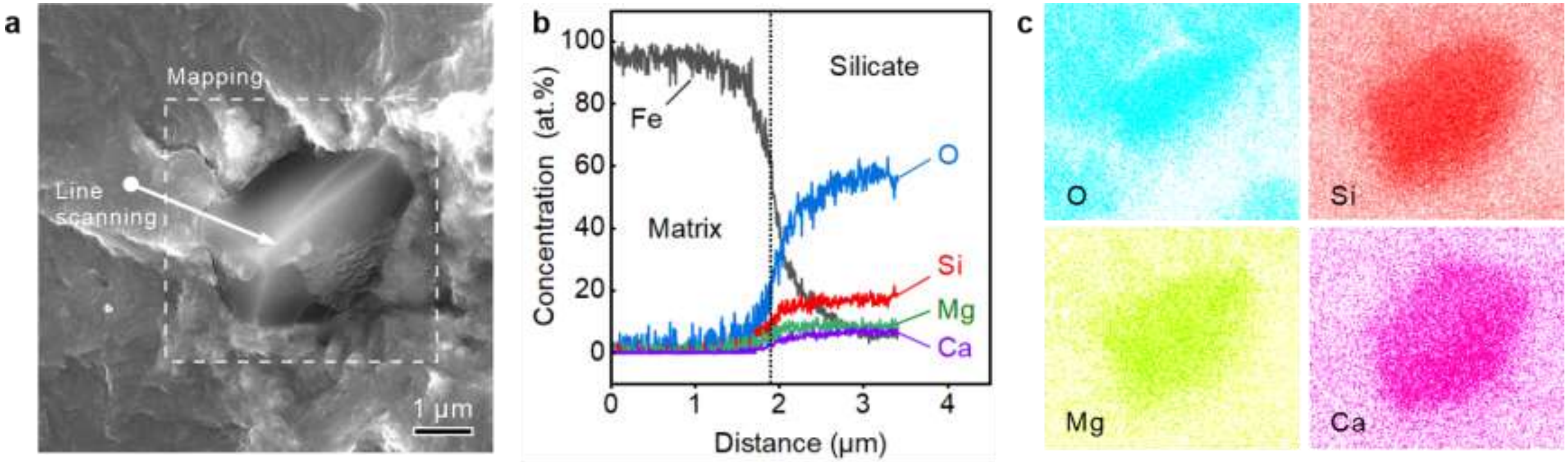

Figure S5. Chemical element analysis of the fatigue cracking source. (a) SEM image of the fatigue cracking source. (b) Line scanning results. (c) Mapping results. Based on the chemical element analysis of the cracking source, the inclusions that cause fatigue cracking have been identified as silicates.

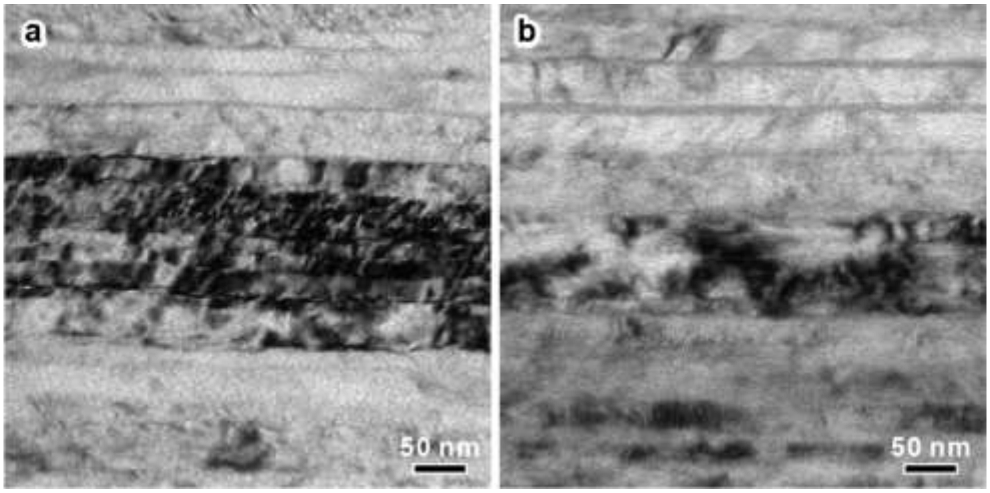

Figure S6. TEM micrographs showing the microstructures. (a) Before and (b) after cyclic loading. There is virtually no change, indicating that the slip band cracking of the matrix is effectively suppressed due to exceptional material homogeneity and ultra-high elastic limit.

Table S1. Analysis of fatigue strength data in Fig. 1g in the main text.

**Stress σ_i (MPa)**	**Level i**	***C*** f_i	***A*** if_i	***B*** i^2f_i
2100	2	2	4	8
2000	1	1	1	1
1900	0	0	0	0
Sum	—	3	5	9

Table S2. The fatigue strength data of different steels.

Material	σ_{UTS} (MPa)	*R*	σ_{fR} (MPa)	$\sigma_{fe0.1}$ (MPa)	σ_{UTS}/ρ (MPa/(g/cm^3))	$\sigma_{fe0.1}/\rho$ (MPa/(g/cm^3))	Ref.
DP980	1095	0.1	611	611	139.49	77.83	[3]
42CrMo4	1050	0.1	970	970	133.76	123.57	[4]
TRIP700	689	0.1	478	478	87.77	60.89	[5]
	780	0.1	462	462	99.36	58.85	
SCr430B	605	0.1	525	525	77.07	66.88	[6]
	1375	0.1	900	900	175.16	114.65	
	1343	0.1	875	875	171.08	111.46	
	1068	0.1	750	750	136.05	95.54	
SAE1055	1055	0.1	775	775	134.39	98.73	
	1511	0.1	875	875	192.48	111.46	
	1868	0.1	975	975	237.96	124.20	
	1290	0.1	975	975	164.33	124.20	
	1006	0.1	825	825	128.15	105.10	
TWIP	950	0.1	475	475	121.02	60.51	
	1004	0.1	575	575	127.90	73.25	
	1129	0.1	750	750	143.82	95.54	
	1357	0.1	800	800	172.87	101.91	
TWIP	1004	0.1	800	800	127.90	101.91	[7]
	1129	0.1	750	750	143.82	95.54	
	1357	0.1	575	575	172.87	73.25	
TWIP	1178	0.1	800	800	150.06	101.91	[8]
	1284	0.1	850	850	163.57	108.28	
	1498	0.1	875	875	190.83	111.46	
	1480	0.1	975	975	188.54	124.20	
FP steel	1184	0.1	775	775	150.83	98.73	
	1293	0.1	800	800	164.71	101.91	
	1537	0.1	925	925	195.80	117.83	
PM tool steel	2432	0.1	1538	1538	309.81	195.92	[9]
QP-0.28	1425	0.1	1000	1000	181.53	127.39	[10]
Sinter-hardened steel	790	0.1	422	422	100.64	53.76	[11]
	650	0.1	300	300	82.80	38.22	
	950	0.1	355	355	121.02	45.22	
Maraging steel	2760	0.1	1150	1150	351.59	146.50	[12]
18Ni maraging steel	2000	0.1	1000	1000	254.78	127.39	[13]
316L	633	0.1	444	444	80.64	56.56	[14]
VDSiCr spring	1984	0.1	1266	1266	252.74	161.27	[15]

steel							
UNI50CrV4	1200	0.1	888	888	152.87	113.12	[16]
SK85	2160	0.1	800	800	275.16	101.91	[17]
100Cr6	2476	0.1	1053	1053	315.41	134.14	[18]
	2149	0.1	1100	1100	273.76	140.13	
GCr15	1780	0.1	1333	1333	226.75	169.81	[19]
	1400	0.1	1167	1167	178.34	148.66	
	2510	0.1	1245	1245	319.75	158.60	
	2578	0.1	1215.5	1216	328.41	154.84	
DV-GCr15	2456	0.1	1342	1342	314.47	171.86	[20]
	2593	0.1	1256	1256	332.01	160.76	
	1797	0.1	1369	1369	230.09	175.27	
	1405	0.1	844	844	178.98	107.57	
EAF-GCr15	2273	0.1	551.25	1225	291.04	156.85	[21]
	2534	0.1	579.38	1288	324.46	164.85	
	1804	0.1	635.63	1413	230.99	180.86	
	1466	0.1	548.44	1219	187.71	156.05	
XT-GCr15	2509	0.1	680.63	1513	321.25	193.66	
	2453	0.1	720	1600	314.08	204.87	
	1798	0.1	658.13	1463	230.22	187.26	
	1432	0.1	544.22	1209	183.35	154.85	
TRIP	1850	0.1	1240	1240	235.67	157.96	[22]
	1370	0.1	886	886	174.52	112.87	
4340	1090	0.1	838	838	138.85	106.75	
	1431	0.1	856	856	182.29	109.04	
ESR4340	1856	0.1	963	963	236.43	122.68	
	2194	0.1	1035	1035	279.49	131.85	
300M	2000	0.1	864	864	254.78	110.06	
HP9-4	1355	0.1	972	972	172.61	123.82	
	1870	0.1	1000	1000	238.22	127.39	
X10CrNiMoV12	1001	0.1	778	778	127.52	99.11	[23]
316L	572	-1	180	289	72.87	36.80	[24]
	658	-1	320	446	83.82	56.82	
	745	-1	420	553	94.90	70.39	
316L	760	-1	325	474	96.82	60.42	[25]
316L	654	-1	316	441	83.31	56.24	[26]
316L	573	-1	225	338	72.99	43.04	[27]
316L	748	-1	350	495	95.29	63.03	[28]
316L	576	-1	305	411	73.38	52.42	[29]
	581	-1	390	476	74.01	60.65	

FV520B	1343	-1	350	590	171.08	75.14	[30]
S31803	686	-1	400	519	87.39	66.12	[31]
	955	-1	350	537	121.66	68.43	
Duplex stainless steel	615	-1	340	451	78.34	57.44	[32]
S31803	840	-1	450	604	107.01	76.98	[33]
	686	-1	375	500	87.39	63.64	
1060	908	-1	305	481	115.67	61.21	[34]
20GL	540	-1	275	377	68.79	47.98	[35]
2.25Cr–1Mo	669	-1	410	521	85.22	66.36	[36]
Cr–Mo–Al steels	1060	-1	510	714	135.03	90.91	[37]
	1000	-1	490	681	127.39	86.76	
35CrMo	919	-1	336	516	117.07	65.74	[38]
	745	-1	388	527	94.90	67.12	
34CrNiMo6	1200	-1	500	736	152.87	93.78	[39]
	1200	0	700	730	152.87	93.05	
38MnVS	838	-1	484	630	106.75	80.32	[40]
	915	-1	562	713	116.56	90.87	
	954	-1	598	752	121.53	95.85	
	858	-1	470	626	109.30	79.69	
38MnVS	885	-1	385	559	112.74	71.15	[41]
39MnVS	858	-1	470	626	109.30	79.69	
39NiCrMo3	908	-1	280	452	115.67	57.57	[42]
40NiCrMo7	1292	-1	380	621	164.59	79.13	
38MnSiVS5	970	-1	340	529	123.57	67.38	[43]
	1243	-1	400	638	158.34	81.27	
42CrMo	1460	-1	680	963	185.99	122.67	[44]
	1014	-1	592	768	129.17	97.80	
	1480	-1	717	1001	188.54	127.49	
	1197	-1	653	871	152.48	110.91	
42CrMoVNb	1540	-1	767	1059	196.18	134.97	[45]
42CrMo4	1535	-1	760	1052	195.54	134.04	[46]
4340	1829	-1	693	1053	232.99	134.08	[47]
	1575	-1	634	944	200.64	120.29	
	1386	-1	629	899	176.56	114.53	
	1286	-1	595	845	163.82	107.59	
	2100	-1	656	1055	267.52	134.39	
54SiCr6	1900	-1	700	1073	242.04	136.63	[48]
50CrV4	1460	-1	670	954	185.99	121.51	[49]
50CrV4	1540	-1	713	1012	196.18	128.90	[50]

	1529	-1	703	1000	194.78	127.41	
	1488	-1	498	785	189.55	100.05	
50CrV4	1750	-1	800	1141	222.93	145.29	[51]
54SiCrV6	1729	-1	770	1108	220.25	141.15	
54SiCr6	1743	-1	720	1063	222.04	135.44	
60Si2Mn	1732	-1	600	937	220.64	119.33	[48]
60Si2CrV	2366	-1	750	1201	301.40	153.03	
60Si2CrV	2365	-1	768	1222	301.27	155.64	[52]
	1750	-1	392	684	222.93	87.12	
	1630	-1	483	788	207.64	100.38	
60Si2Cr	1753	-1	650	994	223.31	126.62	[53]
60Si2CrV	1954	-1	750	1134	248.92	144.52	
60Si2Mn	1813	-1	650	1004	230.96	127.94	
60Si2Mn	2182	-1	650	1059	277.96	134.89	
GCr15VM	1785	-1	725	1077	227.39	137.15	
GCr15ER	1700	-1	775	1106	216.56	140.89	
GCr15RES-T240	2453	-1	1103	1582	328.41	182.44	[54]
GCr15RES-T160	2509	-1	1073	1566	319.62	199.48	
GCr15GER-T160	2578	-1	928	1432	312.48	201.5	
CFB/M-Nb	1640	-1	525	839	208.92	106.82	[55]
	1628	-1	625	945	207.39	120.42	
JIS-SUP7 spring steel	1736	-1	780	1119	221.15	142.53	[56]
EU20SiNb	1640	-1	900	1197	208.92	152.49	[57]
Cr–Mo alloy steel	1408	-1	550	827	179.36	105.38	[58]
	1406	-1	527	803	179.11	102.31	
	1366	-1	605	872	174.01	111.12	
	1363	-1	544	813	173.63	103.51	
MN–Si–Cr–C	1688	-1	900	1211	215.03	154.26	[59]
1Cr-1Mo-0.25V	776	-1	375	524	98.85	66.74	[60]
F22 alloy steel	780	-1	350	502	99.36	63.99	[61]
AISI 329 LN	707	-1	360	493	90.06	62.82	[62]
SUH660	1065	-1	280	471	135.67	59.99	[63]
S31803	840	-1	450	604	107.01	76.98	[64]
	768	-1	350	500	97.83	63.64	
0.5% carbon steel	1600	-1	680	995	203.82	126.69	[65]

B/M steel	1384	-1	760	1011	176.31	128.74	[66]
	1411	-1	625	901	179.75	114.79	
SUP7-T450 spring steel	1586	-1	850	1141	202.04	145.39	[67]
SUJ2	2238	-1	800	1237	285.10	157.61	[68]
GCr15	2300	-1	900	1353	292.99	172.35	[69]
100Cr6	2387	-1	880	1348	304.08	171.73	[70]
18Ni maraging steel	1970	-1	698	1082	250.96	137.88	[71]
	1780	-1	665	1015	226.75	129.24	
	1600	-1	595	909	203.82	115.80	
	1340	-1	555	819	170.70	104.31	
18Ni maraging steel	1939	-1	595	962	247.01	122.49	[72]
	1838	-1	685	1046	234.14	133.23	
	1567	-1	638	947	199.62	120.60	
	1358	-1	588	854	172.99	108.85	
Fe9Mn3Ni1.4Al	1100	-1	400	615	140.13	78.39	[73]

Table S3. The fatigue strength data of additive manufactured Ti-6Al-4V alloy at R = 0.1.

σ_{UTS} (MPa)	$\sigma_{f0.1}$ (MPa)	σ_{UTS}/ρ (MPa/(g/cm^3))	$\sigma_{f0.1}/\rho$ (MPa/(g/cm^3))	Ref.
1200	270	266.08	59.87	[74]
1160	550	257.21	121.95	
1219	550	270.29	121.95	[75]
1280	400	283.81	88.69	[76]
1025	425	227.27	94.24	[77]
937	230	207.74	51.00	[78]
1310	350	290.47	77.61	[79]
925	575	205.10	127.49	
1225	250	271.60	55.43	[80]
974	280	216.01	62.08	
1090	344	241.69	76.27	[81]
1267	430	280.93	95.34	[82]
973	350	215.74	77.61	[83]
991	500	219.73	110.86	[84]
950	700	210.64	155.21	[85]
860	600	190.69	133.04	
1170	500	259.42	110.86	[86]
1108	600	245.68	133.04	[87]
1237	350	274.28	77.61	[88]
1148	300	254.55	66.52	
1257	450	278.71	99.78	
1012	475	224.39	105.32	
1011	600	224.17	133.04	
972	275	215.52	60.98	[89]
1165	490	258.31	108.65	
997	610	221.06	135.25	
1070	200	237.25	44.35	[90]
1030	570	228.38	126.39	
910	460	201.77	102.00	[91]
836	580	185.37	128.60	

816	545	180.93	120.84	[92]
978	600	216.85	133.04	[93]
859	610	190.47	135.25	[94]
842	400	186.70	88.69	
1038	300	230.16	66.52	[95]
1055	788	233.92	174.72	[96]
1228	978	272.28	216.85	[97]
1270	476	281.6	105.54	
977	676	216.63	149.89	
1178	852	261.2	188.91	

Table S4. The fatigue strength data of forged Ti-6Al-4V alloy at R = 0.1.

σ_{UTS} (MPa)	$\sigma_{f0.1}$ (MPa)	σ_{UTS}/ρ (MPa/(g/cm^3))	$\sigma_{f0.1}/\rho$ (MPa/(g/cm^3))	Ref.
1098	550	243.46	121.95	[98]
1053	675	233.48	149.67	
1080	750	239.47	166.30	
917	550	203.33	121.95	
908	600	201.33	133.04	
919	550	203.77	121.95	
978	580	216.85	128.60	[99]
1055	550	233.92	121.95	
980	635	217.29	140.80	[100]
945	750	209.53	166.30	[76]
973	625	215.74	138.58	[101]
943	750	209.09	166.30	[102]
978	580	216.85	128.60	[103]
979	640	217.07	141.91	[104]
1072	500	237.69	110.86	[105]
1058	700	234.59	155.21	[106]
978	600	216.85	133.04	[107]
978	455	216.85	100.89	[108]
978	640	216.85	141.91	
988	835	219.07	185.14	[109]
943	600	209.09	133.04	
965	600	213.97	133.04	[110]
1040	830	230.60	184.04	
1200	722	266.08	160.09	[111]
920	500	203.99	110.86	[112]
1026	661	227.49	146.66	[113]
896	424	198.68	94.03	
1060	616	235.08	136.50	
1183	724	262.38	160.55	
941	474	208.58	105.12	[114]

988	545	219.03	120.88	
985	725	218.40	160.75	[115]

Table S5. The fatigue strength data of other titanium alloys.

Material	σ_{UTS} (MPa)	*R*	σ_{fR} (MPa)	$\sigma_{fe0.1}$ (MPa)	σ_{UTS}/ρ (MPa/(g/cm^3))	$\sigma_{fe0.1}/\rho$ (MPa/(g/cm^3))	Ref.
Ti-4Al-2.5V	1010	0.1	700	700	223.95	155.21	[116]
	1100	0.1	800	800	243.90	177.38	
Ti-6Al-2Zr-1Mo-1V	977	0.1	725	725	216.63	160.75	[117]
	967	0.1	500	500	214.41	110.86	
Ti-5Al-7.5V	1324	0.1	720	720	293.57	159.65	[118]
Ti-6242S	1072	0.1	520	520	237.69	115.30	[106]
	1094	0.1	720	720	242.57	159.65	
	1300	0.1	750	750	288.25	166.30	
	1190	0.1	650	650	263.86	144.12	
	1160	0.1	725	725	257.21	160.75	
	1053	0.1	700	700	233.48	155.21	
TC11	960	0.1	717	717	212.86	158.98	[119]
TC12	1083	0.1	472	472	240.13	104.66	[120]
Ti811	928	0.1	400	400	205.76	88.69	[121]
Ti60	1044	0.1	320	320	231.49	70.95	[122]
Ti-15-3	1496	0.1	875	875	331.71	194.01	[123]
	1206	0.1	750	750	267.41	166.30	
	1365	0.1	775	775	302.66	171.84	
Ti-600	1000	0.1	475	475	221.73	105.32	[124]
Ti-8823	1315	0.1	930	930	291.57	206.21	[125]
	1210	0.1	880	880	268.29	195.12	
Ti–10V–2Fe–3Al	1100	0.1	850	850	243.90	188.47	[126]
	1317	0.1	905	905	292.02	200.67	
	1330	0.1	790	790	294.90	175.17	
Ti-1023	1270	0.1	825	825	281.60	182.93	[127]
Ti-55511	1172	0.1	810	810	259.87	179.60	[128]
	1155	0.1	795	795	256.10	176.27	
	1126	0.1	750	750	249.67	166.30	
	1286	0.1	880	880	285.14	195.12	
	1241	0.1	855	855	275.17	189.58	
β-CEZ	1275	0.1	600	600	282.71	133.04	[129]
Ti-55511	1215	0.06	880	890	269.40	197.44	[130]
	1199	0.06	780	792	265.85	175.56	
	1141	0.06	730	741	252.99	164.38	
	1133	0.06	650	662	251.22	146.79	
BT25	999	0.06	590	600	221.51	133.14	[131]
	1006	0.06	573	584	223.06	129.42	

	983	0.06	526	537	217.96	118.98	
Ti-6246	1160	0.05	550	566	257.21	125.42	[132]
Ti-15-3	1275	0.3	655	575	282.71	127.52	[133]
Ti-55531	1118	-1	652	846	247.89	187.57	[134]
TC11	1110	-1	550	761	246.12	168.79	[135]
TC17	1108	-1	615	814	245.68	180.55	[136]
TC17	1193	-1	549.27	781	264.52	173.19	[137]
TC17	1145	-1	620	829	253.88	183.83	[138]
TC17	1141	-1	629	835	252.99	185.17	[139]
TC17	1140	-1	570	786	252.77	174.33	[140]
TC17	1145	-1	590	804	253.88	178.37	[141]
TC17	1078	-1	480	691	239.02	153.16	[142]
TC21	1200	-1	450	686	266.08	152.04	[143]
TC21	1100	-1	525	737	243.90	163.38	[144]
TC21	1300	-1	650	897	288.25	198.79	[145]
	1240	-1	490	734	274.94	162.81	
Ti811	972	-1	620	774	215.52	171.66	[146]
Ti812	928	-1	510	678	205.76	150.32	[147]
	940	-1	540	705	208.43	156.32	
Ti-35Nb-7Zr-5Ta	1020	-1	490	686	226.16	152.12	[148]
	1010	-1	450	647	223.95	143.56	
Ti55531	1118	-1	652	846	247.89	187.57	[149]
Ti-55531	1293	-1	656	900	286.70	199.51	[150]
Ti-55531	1200	-1	652	871	266.08	193.06	[151]
	1293	-1	656	900	286.70	199.51	
IMI 834	1544	-1	575	878	342.35	194.70	[152]
	1125	-1	750	918	249.45	203.63	

Table S6. The fatigue strength data of different aluminium alloys.

Material	σ_{UTS} (MPa)	*R*	σ_{fR} (MPa)	$\sigma_{fe0.1}$ (MPa)	σ_{UTS}/ρ (MPa/(g/cm^3))	$\sigma_{fe0.1}/\rho$ (MPa/(g/cm^3))	Ref.
2050	470	0.1	225	225	174.07	83.33	[153]
	590	0.1	275	275	218.52	101.85	
2024	601	0.1	356	356	222.59	131.85	[154]
2024	500	0.1	240	240	185.19	88.89	[155]
2198	384	0.1	250	250	142.22	92.59	
2024	473	0.1	151	151	175.19	55.93	[156]
5083	310	0.1	157	157	114.81	58.15	[157]
A357	361	0.1	237	237	133.70	87.78	[158]
6056	393	0.1	222	222	145.56	82.22	[159]
6082	356	0.1	244	244	131.85	90.37	
Al–Mg–Sc–Zr	536	0.1	223	223	198.52	82.59	[160]
AlSi10Mg	241	0.1	120	120	89.26	44.44	[161]
	450	0.1	180	180	166.67	66.67	
	447	0.1	160	160	165.56	59.26	
Al–Zn–Mg	370	0.1	180	180	137.04	66.67	[162]
7050	524	0.1	240	240	194.07	88.89	[163]
7085	569	0.1	240	240	210.74	88.89	[164]
7150	530	0.1	240	240	196.30	88.89	[165]
7055	650	0.1	300	300	240.74	111.11	[166]
A356	225	0.1	110	110	83.33	40.74	[167]
7075	415	0.1	191	191	153.70	70.74	
7050	502	0.1	260	260	185.93	96.30	[168]
7050	566	0.1	215	215	209.63	79.63	[169]
	560	0.1	225	225	207.41	83.33	
	513	0.1	250	250	190.00	92.59	
7039	414	0.1	122	122	153.33	45.19	[170]
	376.4	0.1	133	133	139.41	49.26	
	212.7	0.1	94	94	78.78	34.81	
7075	550	0.1	310	310	203.70	114.81	[171]
7055	700	0.1	350	350	259.26	129.63	[172]
7075	624	0.1	320	320	231.11	118.52	[173]
7075	570	0.1	169	169	211.11	62.59	[174]
7075	552	0.1	178	178	204.44	65.93	[175]
	570	0.1	189	189	211.11	70.00	
7075	536	0.1	272	272	198.52	100.74	[176]
	561	0.1	250	250	207.78	92.59	
7075	550	0.2	480	472	203.70	174.99	[177]

	430	0.2	300	289	159.26	107.07	
2524	252	-0.1	360	334	93.33	123.69	[178]
2009	581	0	250	265	215.19	98.19	[179]
2219	470	0	200	212	174.07	78.59	[180]
2A12	443	-1	160	247	164.07	91.36	[181]
2024	501	-1	185	283	185.56	104.91	[182]
2024	583	-1	204	318	215.93	117.60	[183]
2024	460	-1	187	278	170.37	102.82	[184]
	595	-1	230	347	220.37	128.56	
2024	460	-1	100	176	170.37	65.03	[46]
	641	-1	160	272	237.41	100.90	
2024	510	-1	138	230	188.89	85.35	[185]
2098	575	-1	150	253	212.96	93.61	[186]
Al-Cu	550	-1	114	202	203.70	74.86	[187]
	500	-1	84	155	185.19	57.36	
5056	280	-1	105	160	103.70	59.26	[188]
5056	310	-1	160	218	114.81	80.75	[189]
6082	320	-1	100	161	118.52	59.56	[190]
6061	363	-1	140	211	134.44	78.31	[191]
6061	365	-1	85	147	135.19	54.46	[192]
A6061	103	-1	78	90	38.15	33.34	[193]
6061	378	-1	90	155	140.00	57.38	[194]
	332	-1	100	162	122.96	60.16	
6061	379	-1	150	225	140.37	83.21	[195]
6061	336	-1	110	175	124.44	64.66	[196]
6086	485	-1	150	242	179.63	89.59	[197]
Al6MgSc	410	-1	145	225	151.85	83.32	[198]
A356	279	-1	100	155	103.33	57.23	[199]
	308	-1	120	181	114.07	66.91	
A356-T6	280	-1	110	165	103.70	61.17	[200]
A319	275	-1	99.4	153	101.85	56.74	[201]
Al–12Si–4Cu	262.5	-1	125	176	97.22	65.03	[202]
Al-Mg-Sc	375	-1	150	224	138.89	82.92	[203]
AlSi9Cu3	216	-1	75	117	80.00	43.34	[204]
E319	290	-1	85	139	107.41	51.51	[205]
Al–7Si–Mg	308	-1	130	191	114.07	70.58	[206]
	279	-1	110	165	103.33	61.09	
	293	-1	110	168	108.52	62.06	
	279	-1	90	143	103.33	53.13	

	288	-1	80	133	106.67	49.16	
	255	-1	60	104	94.44	38.35	
7075	595	-1	193	307	220.37	113.75	[207]
	515	-1	173	273	190.74	100.94	
7075	565	-1	213	324	209.26	120.01	[208]
7475	500	-1	195	293	185.19	108.69	[209]
7075	440	-1	140	224	162.96	82.96	
2024	350	-1	160	228	129.63	84.48	
7075	545	-1	150	249	201.85	92.38	[210]
7075	600	-1	165	274	222.22	101.64	[211]
	620	-1	158	268	229.63	99.16	
	565	-1	145	245	209.26	90.85	
7N01	425	-1	167	251	157.41	92.85	
	445	-1	167	254	164.81	94.23	
	430	-1	157	241	159.26	89.35	
7075	635	-1	220	343	235.19	127.21	[212]
7075	669	-1	137	243	247.78	90.18	[213]
	664	-1	137	243	245.93	90.05	
	666	-1	111	205	246.67	75.90	
7050	586	-1	198	311	217.04	115.33	[214]
7050	711	-1	310	449	263.33	166.45	[215]
2024	446	-1	190	278	165.19	102.83	[216]
6061	264	-1	120	171	97.78	63.49	
7050	442	-1	185	272	163.70	100.73	

Table S7. The fatigue strength data of different magnesium alloys.

Material	σ_{UTS} (MPa)	*R*	σ_{fR} (MPa)	$\sigma_{fe0.1}$ (MPa)	σ_{UTS}/ρ (MPa/(g/cm^3))	$\sigma_{fe0.1}/\rho$ (MPa/(g/cm^3))	Ref.
AZ31	189	0.1	70	70	105.00	38.89	[217]
AZ31	282	0.1	200	200	156.67	111.11	[218]
AZ31	238	0.1	110	110	132.22	61.11	[219]
AZ61	295	0.1	188	188	163.89	104.44	[220]
	310	0.1	140	140	172.22	77.78	
	281	0.1	148	148	156.11	82.22	
AM60	224	0.1	162	162	124.44	90.00	[221]
AZ31	283	-1	75	126	157.22	69.94	[222]
	250	-1	90	139	138.89	77.16	
AZ31	210	-1	90	131	116.67	72.92	[223]
AZ31	224	-1	50	87	124.44	48.50	[224]
AZ31	294.5	-1	88	143	163.61	79.58	[225]
AZ31	275	-1	120	174	152.78	96.62	[226]
AZ31	295	-1	100	157	163.89	87.29	[227]
AZ31	301	-1	125	184	167.22	102.36	[228]
AZ61	320	-1	110	172	177.78	95.63	
AZ80	336	-1	150	216	186.67	119.81	
AZ61	318	-1	135	198	176.67	109.73	[229]
AZ91	127	-1	70	93	70.56	51.64	[193]
AZ91	360	-1	125	195	200.00	108.34	[230]
AZ91	336	-1	100	163	186.67	90.53	[231]
AZ91	190	-1	45	78	105.56	43.08	[232]
AZ291	212	1	70	0	117.78	0.00	[233]
AZ61	285	-1	160	211	158.33	117.15	
AZ32 alloy	486	-1	80	148	270.00	82.22	[234]
Mg–10Gd–3Y	225	-1	95	139	125.00	77.36	[235]
	333	-1	120	185	185.00	102.85	
ZM61	297	-1	115	173	165.00	96.37	[236]
	340	-1	105	169	188.89	94.11	
MgGdZnZr	276	-1	105	159	153.33	88.49	[237]
NZ30K	257	-1	87	137	142.78	75.97	[238]
NZ30K	250	-1	105	154	138.89	85.66	[239]
	184	-1	85	121	102.22	67.07	
Mg–10Gd–3Y	445	-1	170	258	247.22	143.07	[240]
	344	-1	150	217	191.11	120.80	
Mg-3Nd-0.2Zn	100	-1	55	73	55.56	40.61	[241]

	200	-1	90	129	111.11	71.68	
	175	-1	70	104	97.22	58.04	
Mg–12Gd–3Y	335	-1	117	182	186.11	101.23	[242]
AM-SC1	192	-1	90	127	106.67	70.64	[243]
	189	-1	88	125	105.00	69.24	
	216	-1	98	140	120.00	77.83	
Mg-3Nd-0.2Zn	197	-1	90	128	109.44	71.30	[244]
	290.5	-1	100	156	161.39	86.90	
	173.9	-1	68	102	96.61	56.80	
	240.3	-1	77	123	133.50	68.31	
	190	-1	83	120	105.56	66.80	
	284.3	-1	98	153	157.94	85.12	
	95.7	-1	54	71	53.17	39.46	
	125.8	-1	64	88	69.89	48.72	
	176	-1	73	108	97.78	59.81	
	256.1	-1	89	139	142.28	77.12	
	72.5	-1	49	60	40.28	33.13	
	107	-1	63	81	59.44	45.23	
	284.1	-1	101	156	157.83	86.92	
Mg–10Gd–3Y	445	-1	165	252	247.22	140.18	[245]
	388	-1	110	182	215.56	100.86	
ZN11	269	-1	75	124	149.44	69.06	
Mg–Zn–Y–Zr	309	-1	85	141	171.67	78.53	[246]
WE43A-T6	244.8	-1	75	121	136.00	67.37	[247]
Mg-Zn-Y-Zr	280	-1	110	165	155.56	91.75	[248]
	262	-1	80	129	145.56	71.92	

Table S8. The fatigue strength data of different copper alloys.

Material	σ_{UTS} (MPa)	*R*	σ_{fR} (MPa)	$\sigma_{fe0.1}$ (MPa)	σ_{UTS}/ρ (MPa/(g/cm^3))	$\sigma_{fe0.1}/\rho$ (MPa/(g/cm^3))	Ref.
Cu	303	0.1	162	162	33.97	18.16	[249]
	231	0.1	110	110	25.90	12.33	
Cu-7Ag	1150	0.1	150	150	128.92	16.82	[250]
Cu	387	-1	140	216	43.39	24.18	[251]
Cu	500	-1	130	219	56.05	24.58	[252]
Cu	387	-1	120	193	43.39	21.68	[253]
Cu	230	-1	95	140	25.78	15.73	[254]
Cu	210	-1	50	86	23.54	9.65	[255]
	330	-1	120	185	37.00	20.70	
	422	-1	100	172	47.31	19.32	
	481	-1	100	177	53.92	19.87	
Cu5 at% Al	257	-1	75	123	28.81	13.77	
	296	-1	120	178	33.18	19.99	
	321	-1	155	217	35.99	24.28	
	573	-1	150	253	64.24	28.31	
Cu-11 at% Al	337	-1	100	163	37.78	18.28	
	448	-1	190	278	50.22	31.17	
	482	-1	210	305	54.04	34.14	
	770	-1	190	324	86.32	36.37	
Cu-15 at% Al	396	-1	110	182	44.39	20.46	
	547	-1	250	356	61.32	39.96	
	592	-1	280	394	66.37	44.20	
	942	-1	200	353	105.61	39.56	
Cu	402	-1	100	170	45.07	19.10	[256]
	232	-1	80	125	26.01	14.02	
Cu-5 at% Zn	433	-1	140	223	48.54	25.00	
	229	-1	75	119	25.67	13.34	
	350	-1	90	152	39.24	17.06	
Cu-11 at% Zn	545	-1	160	262	61.10	29.33	
	614	-1	180	294	68.83	33.01	
Cu–2 at% Be	492	-1	187	284	55.16	31.81	[257]
	675	-1	323	453	75.67	50.77	
	1054	-1	235	410	118.16	46.01	
	1285	-1	183	346	144.06	38.83	

Table S9. The fatigue strength data of different superalloys.

Material	σ_{UTS} (MPa)	R	σ_{fR} (MPa)	$\sigma_{fe0.1}$ (MPa)	σ_{UTS}/ρ (MPa/(g/cm^3))	$\sigma_{fe0.1}/\rho$ (MPa/(g/cm^3))	Ref.
DZ951	1053	0.1	500	500	125.36	59.52	[258]
GH4742	1388	0.1	546	546	165.24	65.00	[259]
Nickel-based single crystal	1157	0.1	650	650	137.74	77.38	[260]
	1095	0.1	640	640	130.36	76.19	
In713C	847	0.1	350	350	100.83	41.67	[261]
GH4169	1374	0.1	744	744	163.57	88.57	[262]
GH4169	1152	0.1	711	711	137.14	84.64	[263]
GH4169	1212	-1	600	831	144.29	98.89	[264]
80A	1312	-1	280	493	156.19	58.75	[265]
	1053	-1	310	507	125.36	60.31	
GH4169	1420	-1	500	777	169.05	92.48	[266]
GH4169	1160	-1	385	609	138.10	72.46	[267]
GH4169	1460	-1	500	783	173.81	93.25	[268]
9CrCo	894	-1	369	545	106.43	64.89	[269]

Table S10. The fatigue strength data of different high-entropy alloys.

Material	σ_{UTS} (MPa)	*R*	σ_{fR} (MPa)	$\sigma_{fe0.1}$ (MPa)	σ_{UTS}/ρ (MPa/(g/cm^3))	$\sigma_{fe0.1}/\rho$ (MPa/(g/cm^3))	Ref.
CrMnFeCoNi	650	-1	220	346	82.80	44.05	[270]
CoCrFeMnNi	626	0.1	280	280	79.75	35.67	[271]
CrMnFeCoNi	585	-1	250	365	74.52	46.49	[272]
Fe42Mn28Cr15Co10Si5	1158	-1	535	760	147.52	96.79	[273]
Fe38.5Mn20Co20Cr15Si5Cu1.5	1126	-1	700	884	143.44	112.60	[274]
As-cast AlCoCrFeNi2.1	1057	-1	383	590	134.65	75.14	[275]
Wrought AlCoCrFeNi2.1	1340	-1	469	730	170.70	92.99	
Al0.3CoCrFeNi	1074	-1	450	661	136.82	84.25	[276]
Al0.5CoCrCuFeNi	1344	0.1	851	851	171.21	108.41	[277]
CoCrFeMnNi	676	-1	190	314	86.11	40.03	[278]
	888	-1	280	449	113.12	57.21	

Supplementary References

1. Izotov VI, Pozdnyakov VA, Luk'yanenko EV *et al.* Influence of the pearlite fineness on the mechanical properties, deformation behavior, and fracture characteristics of carbon steel. *The Physics of Metals and Metallography*. 2007; **103**(5): 519-529. doi: 10.1134/S0031918X07050122
2. Suresh S. *Fatigue of materials*: Cambridge university press, 1998.
3. Xu W, Westerbaan D, Nayak SS *et al.* Tensile and fatigue properties of fiber laser welded high strength low alloy and DP980 dual-phase steel joints. *Mater Des*. 2013; **43**: 373-383. doi: 10.1016/j.matdes.2012.07.017
4. Terres MA, Laalai N, Sidhom H. Effect of nitriding and shot-peening on the fatigue behavior of 42CrMo4 steel: Experimental analysis and predictive approach. *Mater Des*. 2012; **35**: 741-748. doi: 10.1016/j.matdes.2011.09.055
5. Haidemenopoulos GN, Kermanidis AT, Malliaros C *et al.* On the effect of austenite stability on high cycle fatigue of TRIP 700 steel. *Mater Sci Eng, A*. 2013; **573**: 7-11. doi: 10.1016/j.msea.2013.02.015
6. Park SH, Lee CS. Relationship between mechanical properties and high-cycle fatigue strength of medium-carbon steels. *Mater Sci Eng A*. 2017; **690**: 185-194. doi: 10.1016/j.msea.2017.02.080
7. Kim YW, Kim G, Hong S-G *et al.* Energy-based approach to predict the fatigue life behavior of pre-strained Fe–18Mn TWIP steel. *Mater Sci Eng, A*. 2011; **528**(13-14): 4696-4702. doi: 10.1016/j.msea.2011.02.068
8. Song SW, Lee JH, Lee HJ *et al.* Enhancing high-cycle fatigue properties of cold-drawn Fe–Mn–C TWIP steels. *Int J Fatigue*. 2016; **85**: 57-64. doi: 10.1016/j.ijfatigue.2015.12.007

9. Yao J, Qu XH, He XB *et al.* Inclusion-controlled high cycle fatigue behavior of a high V alloyed powder metallurgy cold-working tool steel. *Mater Sci Eng A*. 2011; **528**(12): 4180-4186. doi: 10.1016/j.msea.2011.02.016

10. Diego-Calderón Id, Rodriguez-Calvillo P, Lara A *et al.* Effect of microstructure on fatigue behavior of advanced high strength steels produced by quenching and partitioning and the role of retained austenite. *Mater Sci Eng, A*. 2015; **641**: 215-224. doi: 10.1016/j.msea.2015.06.034

11. D'Armas H, Llanes L, Peñafiel J *et al.* Tempering effects on the tensile response and fatigue life behavior of a sinter-hardened steel. *Mater Sci Eng A*. 2000; **277**(1): 291-296. doi: 10.1016/S0921-5093(99)00533-X

12. Wang W, Yan W, Duan Q *et al.* Study on fatigue property of a new 2.8GPa grade maraging steel. *Mater Sci Eng, A*. 2010; **527**(13-14): 3057-3063. doi: 10.1016/j.msea.2010.02.002

13. Mayer H, Schuller R, Fitzka M *et al.* Very high cycle fatigue of nitrided 18Ni maraging steel sheet. *Int J Fatigue*. 2014; **64**: 140-146. doi: 10.1016/j.ijfatigue.2014.02.003

14. Correa C, Ruiz de Lara L, Díaz M *et al.* Effect of advancing direction on fatigue life of 316L stainless steel specimens treated by double-sided laser shock peening. *Int J Fatigue*. 2015; **79**: 1-9. doi: 10.1016/j.ijfatigue.2015.04.018

15. Mayer H, Schuller R, Karr U *et al.* Cyclic torsion very high cycle fatigue of VDSiCr spring steel at different load ratios. *Int J Fatigue*. 2015; **70**: 322-327. doi: 10.1016/j.ijfatigue.2014.10.007

16. Bertini L, Fontanari V. Fatigue behaviour of induction hardened notched components. *Int J Fatigue*. 1999; **21**(6): 611-617. doi: 10.1016/S0142-1123(99)00019-5

17. Park K, Cho S, Lee K *et al.* Effect of volume fraction of undissolved cementite on the high cycle fatigue properties of high carbon steels. *Int J Fatigue*. 2007; **29**(9-11): 1863-1867. doi: 10.1016/j.ijfatigue.2007.01.001

18. Karsch T, Bomas H, Zoch HW *et al.* Influence of hydrogen content and microstructure on the fatigue behaviour of steel SAE 52100 in the VHCF regime. *Int J Fatigue*. 2014; **60**: 74-89. doi: 10.1016/j.ijfatigue.2013.09.006

19. Wang P, Zhang P, Wang B *et al.* Fatigue cracking criterion of high-strength steels induced by inclusions under high-cycle fatigue. *J Mater Sci Technol*. 2023; **154**: 114-128. doi: 10.1016/j.jmst.2023.02.006

20. Xie ZM, Wang P, Wang B *et al.* Effects of heat treatment on fatigue properties of double vacuum smelting high-carbon chromium-bearing steel. *Adv Eng Mater*. 2022; **24**(10): 2200151. doi: 10.1002/adem.202200151

21. Xu ZK, Wang P, Zhang P *et al.* Fatigue strength optimization of high-strength steels by precisely controlling microstructure and inclusions. *J Mater Sci Technol*. 2025; **230**: 165-176. doi: 10.1016/j.jmst.2025.01.018

22. Olson GB, Chait R, Azrin M *et al.* Fatigue Strength of TRIP Steels. *Metall Trans A*. 1980; **11A**: 1980-1069.

23. Kovacs S, Beck T, Singheiser L. Influence of mean stresses on fatigue life and damage of a turbine blade steel in the VHCF-regime. *Int J Fatigue*. 2013; **49**: 90-99. doi: 10.1016/j.ijfatigue.2012.12.012

24. Huang HW, Wang ZB, Lu J *et al.* Fatigue behaviors of AISI 316L stainless steel with a gradient nanostructured surface layer. *Acta Mater*. 2015; **87**: 150-160. doi: 10.1016/j.actamat.2014.12.057

25. Agarwal N, Kahn H, Avishai A *et al.* Enhanced fatigue resistance in 316L austenitic stainless steel due to low-temperature paraequilibrium carburization. *Acta Mater*. 2007; **55**(16): 5572-5580. doi: 10.1016/j.actamat.2007.06.025

26. Chaves V, Navarro A, Madrigal C. Stage I crack directions under in-phase axial–torsion fatigue loading for AISI 304L stainless steel. *Int J Fatigue*. 2015; **80**: 10-21. doi: 10.1016/j.ijfatigue.2015.05.004

27. Hayashi M, Enomoto K. Effect of preliminary surface working on fatigue strength of type 304 stainless steel at ambient temperature and 288°C in air and pure water environment. *Int J Fatigue*. 2006; **28**(11): 1626-1632. doi: 10.1016/j.ijfatigue.2005.09.016

28. Kamaya M, Kawakubo M. Mean stress effect on fatigue strength of stainless steel. *Int J Fatigue*. 2015; **74**: 20-29. doi: 10.1016/j.ijfatigue.2014.12.006

29. Tokaji K. Fatigue behaviour and fracture mechanism of a 316 stainless steel hardened by carburizing. *Int J Fatigue*. 2004; **26**(5): 543-551. doi: 10.1016/j.ijfatigue.2003.08.024

30. Guo Q, Guo X. Research on high-cycle fatigue behavior of FV520B stainless steel based on intrinsic dissipation. *Mater Des*. 2016; **90**: 248-255. doi: 10.1016/j.matdes.2015.10.103

31. Llanes L, Mateo A, Violan P *et al.* On the high cycle fatigue behavior of duplex stainless steels: Influence of thermal aging. *Mater Sci Eng A*. 1997; **234-236**: 850-852. doi: 10.1016/S0921-5093(97)00359-6

32. Dönges B, Giertler A, Krupp U *et al.* Significance of crystallographic misorientation at phase boundaries for fatigue crack initiation in a duplex stainless steel during high and very high cycle fatigue loading. *Mater Sci Eng, A*. 2014; **589**: 146-152. doi: 10.1016/j.msea.2013.09.098

33. Mateo A, Llanes L, Akdut N *et al.* High cycle fatigue behaviour of a standard duplex stainless steel plate and bar. *Mater Sci Eng A*. 2001; **319-321**: 516-520. doi: 10.1016/S0921-5093(01)01096-6

34. Wagner V, Starke P, Kerscher E *et al.* Cyclic deformation behaviour of railway wheel steels in the very high cycle fatigue (VHCF) regime. *Int J Fatigue*. 2011; **33**(1): 69-74. doi: 10.1016/j.ijfatigue.2010.07.010

35. Mordyuk BN, Prokopenko GI, Volosevich PY *et al.* Improved fatigue behavior of low-carbon steel 20GL by applying ultrasonic impact treatment combined with the electric discharge surface alloying. *Mater Sci Eng, A*. 2016; **659**: 119-129. doi: 10.1016/j.msea.2016.02.036

36. Kobayashi H, Todoroki A, Oomura T *et al.* Ultra-high-cycle fatigue properties and fracture mechanism of modified 2.25Cr–1Mo steel at elevated temperatures. *Int J Fatigue*. 2006; **28**(11): 1633-1639. doi: 10.1016/j.ijfatigue.2005.08.016

37. Zhou C, Wang M, Hui W *et al.* Rotating bending fatigue properties of two case hardening steels after nitriding treatment. *Mater Des*. 2013; **46**: 539-545. doi: 10.1016/j.matdes.2012.08.061

38. Gao C, Yang MQ, Pang JC *et al.* Abnormal relation between tensile and fatigue strengths for a high-strength low-alloy steel. *Mater Sci Eng, A*. 2022; **832**. doi: 10.1016/j.msea.2021.142418

39. Sander M, Müller T, Lebahn J. Influence of mean stress and variable amplitude loading on the fatigue behaviour of a high-strength steel in VHCF regime. *Int J Fatigue*. 2014; **62**: 10-20. doi: 10.1016/j.ijfatigue.2013.04.015

40. Hui W, Chen S, Zhang Y *et al.* Effect of vanadium on the high-cycle fatigue fracture properties of medium-carbon microalloyed steel for fracture splitting connecting rod. *Mater Des*. 2015; **66**: 227-234. doi: 10.1016/j.matdes.2014.10.064

41. Hui W, Zhang Y, Shao C *et al.* Microstructural effects on high-cycle fatigue properties of microalloyed medium carbon steel 38MnVS. *Mater Sci Eng, A*. 2015; **640**: 147-153. doi: 10.1016/j.msea.2015.05.054

42. Bagherifard S, Fernandez-Pariente I, Ghelichi R *et al.* Fatigue behavior of notched steel specimens with nanocrystallized surface obtained by severe shot peening. *Mater Des*. 2013; **45**: 497-503. doi: 10.1016/j.matdes.2012.09.025

43. Sankaran S, Subramanya Sarma V, Padmanabhan KA *et al.* High cycle fatigue behaviour of a multiphase microalloyed medium carbon steel: a comparison between ferrite–pearlite and tempered martensite microstructures. *Mater Sci Eng, A*. 2003; **362**(1-2): 249-256. doi: 10.1016/s0921-5093(03)00583-5

44. Yang ZG, Li SX, Zhang JM *et al.* The fatigue behaviors of zero-inclusion and commercial 42CrMo steels in the super-long fatigue life regime. *Acta Mater*. 2004; **52**(18): 5235-5241. doi: 10.1016/j.actamat.2004.06.031

45. Yang ZG, Yao G, Li GY *et al.* The effect of inclusions on the fatigue behavior of fine-grained high strength 42CrMoVNb steel. *Int J Fatigue*. 2004; **26**(9): 959-966. doi: 10.1016/j.ijfatigue.2004.01.009

46. Stanzl-Tschegg SE, Mayer H. Fatigue and fatigue crack growth of aluminium alloys at very high numbers of cycles. *Int J Fatigue*. 2001; **23**: 231-237. doi: 10.1016/S0142-1123(01)00167-0

47. Pang JC, Li SX, Wang ZG *et al.* General relation between tensile strength and fatigue strength of metallic materials. *Mater Sci Eng A*. 2013; **564**: 331-341. doi: 10.1016/j.msea.2012.11.103

48. Liu YB, Li YD, Li SX *et al.* Prediction of the S–N curves of high-strength steels in the very high cycle fatigue regime. *Int J Fatigue*. 2010; **32**(8): 1351-1357. doi: 10.1016/j.ijfatigue.2010.02.006

49. Shin J-C, Lee S, Hwa Ryu J. Correlation of microstructure and fatigue properties of two high-strength spring steels. *Int J Fatigue*. 1999; **21**(6): 571-579. doi: 10.1016/S0142-1123(99)00010-9

50. Li YD, Yang ZG, Liu YB *et al.* The influence of hydrogen on very high cycle fatigue properties of high strength spring steel. *Mater Sci Eng, A*. 2008; **489**(1-2): 373-379. doi: 10.1016/j.msea.2008.01.089

51. Zhang J, Li S, Yang Z *et al.* Influence of inclusion size on fatigue behavior of high strength steels in the gigacycle fatigue regime. *Int J Fatigue*. 2007; **29**(4): 765-771. doi: 10.1016/j.ijfatigue.2006.06.004

52. Yang ZG, Li SX, Li YD *et al.* Relationship among fatigue life, inclusion size and hydrogen concentration for high-strength steel in the VHCF regime. *Mater Sci Eng A*. 2010; **527**(3): 559-564. doi: 10.1016/j.msea.2009.10.056

53. Liu YB, Yang ZG, Li YD *et al.* On the formation of GBF of high-strength steels in the very high cycle fatigue regime. *Mater Sci Eng, A*. 2008; **497**(1-2): 408-415. doi: 10.1016/j.msea.2008.08.011

54. Wang P, Xu ZK, Zhang P *et al.* The highest fatigue strength for steels. *Acta Mater*. 2025; **289**: 120888. doi: 10.1016/j.actamat.2025.120888

55. Yu Y, Gu JL, Bai BZ *et al.* Very high cycle fatigue mechanism of carbide-free bainite/martensite steel micro-alloyed with Nb. *Mater Sci Eng, A*. 2009; **527**(1-2): 212-217. doi: 10.1016/j.msea.2009.08.024

56. Furuya Y. Notable size effects on very high cycle fatigue properties of high-strength steel. *Mater Sci Eng A*. 2011; **528**(15): 5234-5240. doi: 10.1016/j.msea.2011.03.082

57. Zhao P, Cheng C, Gao G *et al.* The potential significance of microalloying with niobium in governing very high cycle fatigue behavior of bainite/martensite multiphase steels. *Mater Sci Eng, A*. 2016; **650**: 438-444. doi: 10.1016/j.msea.2015.10.044

58. Hui W, Zhang Y, Zhao X *et al.* Very high cycle fatigue properties of Cr–Mo low alloy steel containing V-rich MC type carbides. *Mater Sci Eng, A*. 2016; **651**: 311-320. doi: 10.1016/j.msea.2015.10.124

59. Zhao P, Zhang B, Cheng C *et al.* The significance of ultrafine film-like retained austenite in governing very high cycle fatigue behavior in an ultrahigh-strength MN–SI–Cr–C steel. *Mater Sci Eng A*. 2015; **645**: 116-121. doi: 10.1016/j.msea.2015.07.095

60. Suh C-M, Hwang B-W, Murakami R-I. Behaviors of residual stress and high-temperature fatigue life in ceramic coatings produced by PVD. *Mater Sci Eng A*. 2003; **343**(1): 1-7. doi: 10.1016/S0921-5093(02)00327-1

61. Gaur V, Doquet V, Persent E *et al.* Surface versus internal fatigue crack initiation in steel: Influence of mean stress. *Int J Fatigue*. 2016; **82**: 437-448. doi: 10.1016/j.ijfatigue.2015.08.028

62. Knobbe H, Starke P, Hereñú S *et al.* Cyclic deformation behaviour, microstructural evolution and fatigue life of duplex steel AISI 329 LN. *Int J Fatigue*. 2015; **80**: 81-89. doi: 10.1016/j.ijfatigue.2015.05.002

63. Wu H, Hamada S, Oda Y *et al.* Effect of internal hydrogen on very high cycle fatigue of precipitation-strengthened steel SUH660. *Int J Fatigue*. 2015; **70**: 406-416. doi: 10.1016/j.ijfatigue.2014.07.006

64. Mateo A. Anisotropy effects on the fatigue behaviour of rolled duplex stainless steels. *Int J Fatigue*. 2003; **25**(6): 481-488. doi: 10.1016/s0142-1123(02)00173-1

65. Kang M, Aono Y, Noguchi H. Effect of prestrain on and prediction of fatigue limit in carbon steel. *Int J Fatigue*. 2007; **29**(9-11): 1855-1862. doi: 10.1016/j.ijfatigue.2007.01.024

66. Yu Y, Gu JL, Shou FL *et al.* Competition mechanism between microstructure type and inclusion level in determining VHCF behavior of bainite/martensite dual phase steels. *Int J Fatigue*. 2011; **33**(3): 500-506. doi: 10.1016/j.ijfatigue.2010.10.004

67. Li W, Sakai T, Wakita M *et al.* Influence of microstructure and surface defect on very high cycle fatigue properties of clean spring steel. *Int J Fatigue*. 2014; **60**: 48-56. doi: 10.1016/j.ijfatigue.2013.06.017

68. Akiniwa Y, Miyamoto N, Tsuru H *et al.* Notch effect on fatigue strength reduction of bearing steel in the very high cycle regime. *Int J Fatigue*. 2006; **28**(11): 1555-1565. doi: 10.1016/j.ijfatigue.2005.04.017

69. Li W, Sakai T, Li Q *et al.* Reliability evaluation on very high cycle fatigue property of GCr15 bearing steel. *Int J Fatigue*. 2010; **32**(7): 1096-1107. doi: 10.1016/j.ijfatigue.2009.12.008

70. Mayer H, Haydn W, Schuller R *et al.* Very high cycle fatigue properties of bainitic high carbon–chromium steel under variable amplitude conditions. *Int J Fatigue*. 2009; **31**(8-9): 1300-1308. doi: 10.1016/j.ijfatigue.2009.02.038

71. Xu ZK, Wang B, Zhang P *et al.* A fast evaluation method for fatigue strength of maraging steel: The minimum strength principle. *Mater Sci Eng, A*. 2020; **789**. doi: 10.1016/j.msea.2020.139659

72. Wang B, Zhang P, Duan QQ *et al.* Optimizing the fatigue strength of 18Ni maraging steel through ageing treatment. *Mater Sci Eng, A*. 2017; **707**: 674-688. doi: 10.1016/j.msea.2017.09.107

73. Koyama M, Zhang Z, Wang Mm *et al.* Bone-like crack resistance in hierarchical metastable nanolaminate steels. *Science*. 2017; **355**(6329): 1055-1057. doi: 10.1126/science.aal2766

74. Gerov MV, Vladislavskaya EY, Terent'ev VF *et al.* Fatigue strength of a Ti–6Al–4V alloy produced by selective laser melting. *Russ Metall*. 2017; **2016**(10): 935-941. doi: 10.1134/s0036029516100049

75. Rafi HK, Starr TL, Stucker BE. A comparison of the tensile, fatigue, and fracture behavior of Ti–6Al–4V and 15-5 PH stainless steel parts made by selective laser melting. *Int J Adv Manuf Technol*. 2013; **69**(5-8): 1299-1309. doi: 10.1007/s00170-013-5106-7

76. Xu W, Sun S, Elambasseril J *et al.* Ti-6Al-4V Additively Manufactured by Selective Laser Melting with Superior Mechanical Properties. *JOM*. 2015; **67**(3): 668-673. doi: 10.1007/s11837-015-1297-8

77. Wycisk E, Emmelmann C, Siddique S *et al.* High Cycle Fatigue (HCF) Performance of Ti-6Al-4V Alloy Processed by Selective Laser Melting. *Adv Mater Res*. 2013; **816-817**: 134-139. doi: 10.4028/www.scientific.net/AMR.816-817.134

78. Rekedal K, Liu D. Fatigue life of selective laser melted and hot isostatically pressed Ti-6Al-4V absent of surface machining. In: *56th AIAA/ASCE/AHS/ASC structures, structural dynamics, and materials conference, 2015*.

79. Zhao X, Li S, Zhang M *et al.* Comparison of the microstructures and mechanical properties of Ti–6Al–4V fabricated by selective laser melting and electron beam melting. *Mater Des*. 2016; **95**: 21-31. doi: 10.1016/j.matdes.2015.12.135

80. Jiang Q, Li S, Zhou C *et al.* Effects of laser shock peening on the ultra-high cycle fatigue performance of additively manufactured Ti6Al4V alloy. *Opt Laser Technol*. 2021; **144**. doi: 10.1016/j.optlastec.2021.107391

81. Benedetti M, Fontanari V, Bandini M *et al.* Low- and high-cycle fatigue resistance of Ti-6Al-4V ELI additively manufactured via selective laser melting: Mean stress and defect sensitivity. *Int J Fatigue*. 2018; **107**: 96-109. doi: 10.1016/j.ijfatigue.2017.10.021

82. Hu YN, Wu SC, Withers PJ *et al.* The effect of manufacturing defects on the fatigue life of selective laser melted Ti-6Al-4V structures. *Mater Des*. 2020; **192**. doi: 10.1016/j.matdes.2020.108708

83. Kasperovich G, Hausmann J. Improvement of fatigue resistance and ductility of TiAl6V4 processed by selective laser melting. *J Mater Process Technol*. 2015; **220**: 202-214. doi: 10.1016/j.jmatprotec.2015.01.025

84. Le V-D, Pessard E, Morel F *et al.* Fatigue behaviour of additively manufactured Ti-6Al-4V alloy: The role of defects on scatter and statistical size effect. *Int J Fatigue*. 2020; **140**. doi: 10.1016/j.ijfatigue.2020.105811

85. Brandl E, Leyens C, Palm F. Mechanical Properties of Additive Manufactured Ti-6Al-4V Using Wire and Powder Based Processes. *IOP Conference Series: Materials Science and Engineering*. 2011; **26**. doi: 10.1088/1757-899x/26/1/012004

86. Aguado-Montero S, Navarro C, Vázquez J *et al.* Fatigue behaviour of PBF additive manufactured TI6AL4V alloy after shot and laser peening. *Int J Fatigue*. 2022; **154**. doi: 10.1016/j.ijfatigue.2021.106536

87. Kahlin M, Ansell H, Basu D *et al.* Improved fatigue strength of additively manufactured Ti6Al4V by surface post processing. *Int J Fatigue*. 2020; **134**. doi: 10.1016/j.ijfatigue.2020.105497

88. Gong H, Rafi K, Gu H *et al.* Influence of defects on mechanical properties of Ti–6Al–4V components produced by selective laser melting and electron beam melting. *Mater Des*. 2015; **86**: 545-554. doi: 10.1016/j.matdes.2015.07.147

89. Greitemeier D, Palm F, Syassen F *et al.* Fatigue performance of additive manufactured TiAl6V4 using electron and laser beam melting. *Int J Fatigue*. 2017; **94**: 211-217. doi: 10.1016/j.ijfatigue.2016.05.001

90. Hrabe N, Gnäupel-Herold T, Quinn T. Fatigue properties of a titanium alloy (Ti–6Al–4V) fabricated via electron beam melting (EBM): Effects of internal defects and residual stress. *Int J Fatigue*. 2017; **94**: 202-210. doi: 10.1016/j.ijfatigue.2016.04.022

91. Shui X, Yamanaka K, Mori M *et al.* Effects of post-processing on cyclic fatigue response of a titanium alloy additively manufactured by electron beam melting. *Mater Sci Eng, A*. 2017; **680**: 239-248. doi: 10.1016/j.msea.2016.10.059

92. Suo H, Chen Z, Liu J *et al.* Microstructure and Mechanical Properties of Ti-6Al-4V by Electron Beam Rapid Manufacturing. *Rare Metal Materials and Engineering*. 2014; **43**(4): 780-785. doi: 10.1016/s1875-5372(14)60083-7

93. Mohammadhosseini A, Fraser D, Masood SH *et al.* Microstructure and mechanical properties of Ti–6Al–4V manufactured by electron beam melting process. *Mater Res Innovations*. 2013; **17**(sup2): s106-s112. doi: 10.1179/1432891713z.000000000302

94. Biswal R, Zhang X, Syed AK *et al.* Criticality of porosity defects on the fatigue performance of wire + arc additive manufactured titanium alloy. *Int J Fatigue*. 2019; **122**: 208-217. doi: 10.1016/j.ijfatigue.2019.01.017

95. Sterling AJ, Torries B, Shamsaei N *et al.* Fatigue behavior and failure mechanisms of direct laser deposited Ti–6Al–4V. *Mater Sci Eng, A*. 2016; **655**: 100-112. doi: 10.1016/j.msea.2015.12.026

96. Alegre JM, Díaz A, García R *et al.* Effect of HIP post-processing at 850 °C/200 MPa in the fatigue behavior of Ti-6Al-4V alloy fabricated by Selective Laser Melting. *Int J Fatigue*. 2022; **163**: 107097. doi: 10.1016/j.ijfatigue.2022.107097

97. Qu Z, Zhang ZJ, Liu R *et al.* High fatigue resistance in a titanium alloy via near-void-free 3D printing. *Nature*. 2024; **626**(8001): 999-1004. doi: 10.1038/s41586-024-07048-1

98. Hagiwara M, Kitaura T, Ono Y *et al.* High Cycle Fatigue Properties of a Minor Boron-Modified Ti–6Al–4V Alloy. *Mater Trans*. 2012; **53**(8): 1486-1494. doi: 10.2320/matertrans.M2012104

99. Nalla RK, Boyce BL, Campbell JP *et al.* Influence of microstructure on high-cycle fatigue of Ti-6Al-4V: Bimodal vs. lamellar structures. *Metall Mater Trans A*. 2002; **33**(13): 899-918. doi: 10.1007/s11661-002-1023-3

100. Golden PJ, John R, Porter WJ. Investigation of variability in fatigue crack nucleation and propagation in alpha+beta Ti-6Al-4V. *Procedia Engineering*. 2010; **2**(1): 1839-1847. doi: 10.1016/j.proeng.2010.03.198

101. Mower TM. Degradation of titanium 6Al–4V fatigue strength due to electrical discharge machining. *Int J Fatigue*. 2014; **64**: 84-96. doi: 10.1016/j.ijfatigue.2014.02.018

102. Yoshinaka F, Nakamura T, Nakayama S *et al.* Non-destructive observation of internal fatigue crack growth in Ti–6Al–4V by using synchrotron radiation μCT imaging. *Int J Fatigue*. 2016; **93**: 397-405. doi: 10.1016/j.ijfatigue.2016.05.028

103. Bellows RS, Muju S, Nicholas T. Validation of the step test method for generating Haigh diagrams for Ti-6Al-4V. *Int J Fatigue*. 1999; **21**(7): 687-697. doi: 10.1016/S0142-1123(99)00032-8

104. Jeong D, Kwon Y, Goto M *et al.* High cycle fatigue and fatigue crack propagation behaviors of β-annealed Ti-6Al-4V alloy. *Int J Mech Eng*. 2017; **12**(1). doi: 10.1186/s40712-016-0069-8

105. Seo W, Jeong D, Lee D *et al.* Effects of cooling rate and stabilization annealing on fatigue behavior of β-processed Ti-6Al-4V alloys. *Met Mater Int*. 2017; **23**(4): 648-659. doi: 10.1007/s12540-017-6730-9

106. Hagiwara M, Kitashima T, Emura S. Relationship between microstructures, facet morphologies at the high-cycle fatigue (HCF) crack initiation site, and HCF strength in Ti-6242S. *Mater Sci Eng, A*. 2018; **727**: 43-50. doi: 10.1016/j.msea.2018.04.043

107. Jha SK, Szczepanski CJ, Golden PJ *et al.* Characterization of fatigue crack-initiation facets in relation to lifetime variability in Ti–6Al–4V. *Int J Fatigue*. 2012; **42**: 248-257. doi: 10.1016/j.ijfatigue.2011.11.017

108. Prevéy PS, Shepard MJ, Smith PR. The effect of low plasticity burnishing (LPB) on the HCF performance and FOD resistance of Ti-6AI-4V. In: *Proceedings 6th national turbine engine high cycle fatigue conference, 2001*.

109. Oguma H, Nakamura T. The effect of microstructure on very high cycle fatigue properties in Ti–6Al–4V. *Scr Mater*. 2010; **63**(1): 32-34. doi: 10.1016/j.scriptamat.2010.02.043

110. Akahori T, Niinomi M, Fukunaga K *et al.* Effects of microstructure on the short fatigue crack initiation and propagation characteristics of biomedical alpha/beta titanium alloys. *Metall Mater Trans A*. 2000; **31**(8): 1949-1958. doi: 10.1007/s11661-000-0222-z

111. Chan KS. Roles of microstructure in fatigue crack initiation. *Int J Fatigue*. 2010; **32**(9): 1428-1447. doi: 10.1016/j.ijfatigue.2009.10.005

112. Oh J, Lee JG, Kim NJ *et al.* Effects of thickness on fatigue properties of investment cast Ti-6Al-4V alloy plates. *J Mater Sci*. 2004; **39**(2): 587-591. doi: 10.1023/B:JMSC.0000011515.84569.ec

113. MMPDS. *Metallic Materials Properties Development and Standardization (MMPDS-11)*: Battelle Memorial Institute, Columbus, Ohio, 2016.

114.Ivanova SG, Biederman RR, Sisson RD. Investigation of fatigue crack initiation in Ti-6Al-4V during tensile-tensile fatigue. *J Mater Eng Perform*. 2002; **11**(2): 226-231. doi: 10.1361/105994902770344312

115.Zuo JH, Wang ZG, Han EH. The effect of ion irradiation on the tensile and fatigue properties of Ti–6Al–4V alloy. *Mater Sci Eng, A*. 2010; **527**(15): 3396-3401. doi: 10.1016/j.msea.2010.02.014

116.Srivatsan TS, Kuruvilla M, Park L. A study at understanding the mechanisms governing the high cycle fatigue and final fracture behavior of the titanium alloy: Ti–4Al–2.5V. *Mater Sci Eng, A*. 2010; **527**(3): 435-448. doi: 10.1016/j.msea.2009.09.012

117.Li S, Xiong B, Hui S *et al.* Comparison of the fatigue and fracture of Ti–6Al–2Zr–1Mo–1V with lamellar and bimodal microstructures. *Mater Sci Eng, A*. 2007; **460-461**: 140-145. doi: 10.1016/j.msea.2007.02.064

118.Wu Z, Kou H, Chen N *et al.* Crack initiation mechanism in a high-strength Ti-5Al-7.5V alloy subjected to high cycle fatigue loading. *Eng Fail Anal*. 2023; **148**. doi: 10.1016/j.engfailanal.2023.107201

119.Wu Y, Xiong Y, Liu W *et al.* Effect of supersonic fine particle bombardment on microstructure and fatigue properties of Ti-6.5Al-3.5Mo-1.5Zr-0.3Si titanium alloy at different temperatures. *Surf Coat Technol*. 2021; **421**. doi: 10.1016/j.surfcoat.2021.127473

120. Zhao Xh, Xue Gl, Liu Y. Gradient crystalline structure induced by ultrasonic impacting and rolling and its effect on fatigue behavior of TC11 titanium alloy. *Results Phys*. 2017; **7**: 1845-1851. doi: 10.1016/j.rinp.2017.05.026

121. Yang K, Zhong B, Huang Q *et al.* Stress ratio effect on notched fatigue behavior of a Ti-8Al-1Mo-1V alloy in the very high cycle fatigue regime. *Int J Fatigue*. 2018; **116**: 80-89. doi: 10.1016/j.ijfatigue.2018.05.032

122. He R, Peng H, Liu F *et al.* Crack Initiation Mechanism and Life Prediction of Ti60 Titanium Alloy Considering Stress Ratios Effect in Very High Cycle Fatigue Regime. *Materials*. 2022; **15**(8). doi: 10.3390/ma15082800

123. Boyer RR, Rack HJ, Venkatesh V. The influence of thermomechanical processing on the smooth fatigue properties of Ti–15V–3Cr–3Al–3Sn. *Mater Sci Eng A*. 1998; **243**(1): 97-102. doi: 10.1016/S0921-5093(97)00785-5

124. Liying Z, Yongqing Z, Quan H *et al.* High cycle fatigue property of Ti-600 alloy at ambient temperature. *J Alloys Compd*. 2011; **509**(5): 2081-2086. doi: 10.1016/j.jallcom.2010.10.144

125. Chait R, DeSisto TS. The influence of grain size on the high cycle fatigue crack initiation of a metastable beta Ti alloy. *Metall Trans A*. 1977; **8**(6): 1017-1020. doi: 10.1007/BF02661593

126. Wu Y, Liu J, Wang H *et al.* Effect of stress ratio on very high cycle fatigue properties of Ti-10V-2Fe-3Al alloy with duplex microstructure. *J Mater Sci Technol*. 2018; **34**(7): 1189-1195. doi: 10.1016/j.jmst.2017.11.036

127. Jha SK, Ravi Chandran KS. An unusual fatigue phenomenon: duality of the S–N fatigue curve in the β-titanium alloy Ti–10V–2Fe–3Al. *Scr Mater*. 2003; **48**(8): 1207-1212. doi: 10.1016/s1359-6462(02)00565-1

128. Shi X, Zeng W, Xue S *et al.* The crack initiation behavior and the fatigue limit of Ti–5Al–5Mo–5V–1Cr–1Fe titanium alloy with basket-weave microstructure. *J Alloys Compd*. 2015; **631**: 340-349. doi: 10.1016/j.jallcom.2015.01.077

129. Peters JO, Lütjering G. Comparison of the fatigue and fracture of α+β and β titanium alloys. *Metall Mater Trans A*. 2001; **32**(11): 2805-2818. doi: 10.1007/s11661-001-1031-8

130. Wu GQ, Shi CL, Sha W *et al.* Microstructure and high cycle fatigue fracture surface of a Ti–5Al–5Mo–5V–1Cr–1Fe titanium alloy. *Mater Sci Eng, A*. 2013; **575**: 111-118. doi: 10.1016/j.msea.2013.03.047

131. Zhang S, Zeng W, Zhao Q *et al.* High cycle fatigue of isothermally forged Ti-6.5Al-2.2Mo-2.2Zr-1.8Sn-0.7W-0.2Si with different microstructures. *J Alloys Compd*. 2016; **689**: 114-122. doi: 10.1016/j.jallcom.2016.07.277

132. Szczepanski CJ, Jha SK, Larsen JM *et al.* Microstructural influences on very-high-cycle fatigue-crack initiation in Ti-6246. *Metall Mater Trans A*. 2008; **39**(12): 2841-2851. doi: 10.1007/s11661-008-9633-z

133. Santhosh R, Geetha M, Saxena VK *et al.* Effect of duplex aging on microstructure and mechanical behavior of beta titanium alloy Ti–15V–3Cr–3Al–3Sn under unidirectional and cyclic loading conditions. *Int J Fatigue*. 2015; **73**: 88-97. doi: 10.1016/j.ijfatigue.2014.12.005

134. Zhang Z, Huang C, Xu Z *et al.* Influence of notch root radius on high cycle fatigue properties and fatigue crack initiation behavior of Ti-55531 alloy with a multilevel lamellar microstructure. *J Mater Res Technol*. 2023; **24**: 6293-6311. doi: 10.1016/j.jmrt.2023.04.211

135. Shi H, Liu D, Jia T *et al.* Effect of the ultrasonic surface rolling process and plasma electrolytic oxidation on the hot salt corrosion fatigue behavior of TC11 alloy. *Int J Fatigue*. 2023; **168**. doi: 10.1016/j.ijfatigue.2022.107443

136. Li G, Sun C. High-temperature failure mechanism and defect sensitivity of TC17 titanium alloy in high cycle fatigue. *J Mater Sci Technol*. 2022; **122**: 128-140. doi: 10.1016/j.jmst.2022.01.010

137. Jinlong W, Wenjie P, Jing Y *et al.* Fatigue evaluation of TC17 titanium alloy shaft with surface scratch based on FEA and fracture mechanics. *Eng Fail Anal*. 2020; **117**. doi: 10.1016/j.engfailanal.2020.104961

138. Shi H, Liu D, Pan Y *et al.* Effect of shot peening and vibration finishing on the fatigue behavior of TC17 titanium alloy at room and high temperature. *Int J Fatigue*. 2021; **151**. doi: 10.1016/j.ijfatigue.2021.106391

139. Chi W, Wang W, Xu W *et al.* Effects of defects on fatigue behavior of TC17 titanium alloy for compressor blades: Crack initiation and modeling of fatigue strength. *Eng Fract Mech*. 2022; **259**. doi: 10.1016/j.engfracmech.2021.108136

140. Jiao S, Gao C, Cheng L *et al.* A Very High-Cycle Fatigue Test and Fatigue Properties of TC17 Titanium Alloy. *J Mater Eng Perform*. 2016; **25**(3): 1085-1093. doi: 10.1007/s11665-016-1930-x

141. Sun C, Wu H, Chi W *et al.* Nanograin formation and cracking mechanism in Ti alloys under very high cycle fatigue loading. *Int J Fatigue*. 2023; **167**. doi: 10.1016/j.ijfatigue.2022.107331

142. Liu F, Chen Y, He C *et al.* Tensile and very high cycle fatigue behaviors of a compressor blade titanium alloy at room and high temperatures. *Mater Sci Eng, A*. 2021; **811**. doi: 10.1016/j.msea.2021.141049

143. Tan C, Sun Q, Xiao L *et al.* Cyclic deformation and microcrack initiation during stress controlled high cycle fatigue of a titanium alloy. *Mater Sci Eng, A*. 2018; **711**: 212-222. doi: 10.1016/j.msea.2017.11.019

144. Nie B, Zhao Z, Chen D *et al.* Effect of Basketweave Microstructure on Very High Cycle Fatigue Behavior of TC21 Titanium Alloy. *Metals*. 2018; **8**(6). doi: 10.3390/met8060401

145. Tan C, Sun Q, Zhang G *et al.* High-cycle fatigue of a titanium alloy: the role of microstructure in slip irreversibility and crack initiation. *J Mater Sci*. 2020; **55**(26): 12476-12487. doi: 10.1007/s10853-020-04845-7

146. Gao T, Xue H, Sun Z *et al.* Micromechanisms of crack initiation of a Ti-8Al-1Mo-1V alloy in the very high cycle fatigue regime. *Int J Fatigue*. 2021; **150**. doi: 10.1016/j.ijfatigue.2021.106314

147. Yang K, Huang Q, Zhong B *et al.* Influence of the volume content of α + β colonies on the very high cycle fatigue behavior of a titanium alloy. *Fatigue Fract Eng Mater Struct*. 2021; **44**(10): 2643-2658. doi: 10.1111/ffe.13520

148. Long M, Crooks R, Rack HJ. High-cycle fatigue performance of solution-treated metastable-β titanium alloys. *Acta Mater*. 1999; **47**(2): 661-669. doi: 10.1016/S1359-6454(98)00343-7

149. Huang C, Zhao Y, Xin S *et al.* High cycle fatigue behavior of Ti–5Al–5Mo–5V–3Cr–1Zr titanium alloy with lamellar microstructure. *Mater Sci Eng, A*. 2017; **682**: 107-116. doi: 10.1016/j.msea.2016.11.014

150. Huang C, Zhao Y, Xin S *et al.* High cycle fatigue behavior of Ti–5Al–5Mo–5V–3Cr–1Zr titanium alloy with bimodal microstructure. *J Alloys Compd*. 2017; **695**: 1966-1975. doi: 10.1016/j.jallcom.2016.11.031

151. Huang C, Zhao Y, Xin S *et al.* Effect of microstructure on high cycle fatigue behavior of Ti–5Al–5Mo–5V–3Cr–1Zr titanium alloy. *Int J Fatigue*. 2017; **94**: 30-40. doi: 10.1016/j.ijfatigue.2016.09.005

152. Herasymchuk OM. Nonlinear relationship between the fatigue limit and quantitative parameters of material microstructure. *Int J Fatigue*. 2011; **33**(4): 649-659. doi: 10.1016/j.ijfatigue.2010.11.015

153. Guérin M, Alexis J, Andrieu E *et al.* Corrosion-fatigue lifetime of Aluminium–Copper–Lithium alloy 2050 in chloride solution. *Mater Des*. 2015; **87**: 681-692. doi: 10.1016/j.matdes.2015.08.003

154. Htoo AT, Miyashita Y, Otsuka Y *et al.* Variation of local stress ratio and its effect on notch fatigue behavior of 2024-T4 aluminum alloy. *Int J Fatigue*. 2016; **88**: 19-28. doi: 10.1016/j.ijfatigue.2016.03.001

155. Alexopoulos ND, Migklis E, Stylianos A *et al.* Fatigue behavior of the aeronautical Al–Li (2198) aluminum alloy under constant amplitude loading. *Int J Fatigue*. 2013; **56**: 95-105. doi: 10.1016/j.ijfatigue.2013.07.009

156. Mayer H, Schuller R, Fitzka M. Fatigue of 2024-T351 aluminium alloy at different load ratios up to 1010 cycles. *Int J Fatigue*. 2013; **57**: 113-119. doi: 10.1016/j.ijfatigue.2012.07.013

157. Sidhom N, Laamouri A, Fathallah R *et al.* Fatigue strength improvement of 5083 H11 Al-alloy T-welded joints by shot peening: experimental characterization and predictive approach. *Int J Fatigue*. 2005; **27**(7): 729-745. doi: 10.1016/j.ijfatigue.2005.02.001

158. Dezecot S, Brochu M. Microstructural characterization and high cycle fatigue behavior of investment cast A357 aluminum alloy. *Int J Fatigue*. 2015; **77**: 154-159. doi: 10.1016/j.ijfatigue.2015.03.004

159. Schwerdt D, Pyttel B, Berger C. Fatigue strength and failure mechanisms of wrought aluminium alloys in the VHCF-region considering material and component relevant influencing factors. *Int J Fatigue*. 2011; **33**(1): 33-41. doi: 10.1016/j.ijfatigue.2010.05.008

160. Qin Z, Kang N, El Mansori M *et al.* Anisotropic high cycle fatigue property of Sc and Zr-modified Al-Mg alloy fabricated by laser powder bed fusion. *Addit Manuf*. 2022; **49**. doi: 10.1016/j.addma.2021.102514

161. Baek M-S, Kreethi R, Park T-H *et al.* Influence of heat treatment on the high-cycle fatigue properties and fatigue damage mechanism of selective laser melted AlSi10Mg alloy. *Mater Sci Eng, A*. 2021; **819**. doi: 10.1016/j.msea.2021.141486

162. Jiang K-D, Zhang Z, Zhu W-B *et al.* Influence of V additions on microstructures, tensile and fatigue properties of Al–Zn–Mg alloys. *Mater Sci Eng, A*. 2022; **829**. doi: 10.1016/j.msea.2021.142184

163. Luong H, Hill MR. The effects of laser peening and shot peening on high cycle fatigue in 7050-T7451 aluminum alloy. *Mater Sci Eng, A*. 2010; **527**(3): 699-707. doi: 10.1016/j.msea.2009.08.045

164. Luong H, Hill MR. The effects of laser peening on high-cycle fatigue in 7085-T7651 aluminum alloy. *Mater Sci Eng, A*. 2008; **477**(1-2): 208-216. doi: 10.1016/j.msea.2007.05.024

165. Curtis SA, Romero JS, de los Rios ER *et al.* Predicting the interfaces between fatigue crack growth regimes in 7150-T651 aluminium alloy using the fatigue damage map. *Mater Sci Eng A*. 2003; **344**(1): 79-85. doi: 10.1016/S0921-5093(02)00416-1

166. Srivatsan TS, Anand S, Sriram S *et al.* The high-cycle fatigue and fracture behavior of aluminum alloy 7055. *Mater Sci Eng A*. 2000; **281**(1): 292-304. doi: 10.1016/S0921-5093(99)00716-9

167. Peyre P, Fabbro R, Merrien P *et al.* Laser shock processing of aluminium alloys. Application to high cycle fatigue behaviour. *Mater Sci Eng A*. 1996; **210**(1): 102-113. doi: 10.1016/0921-5093(95)10084-9

168. Carvalho ALM, Voorwald HJC. Influence of shot peening and hard chromium electroplating on the fatigue strength of 7050-T7451 aluminum alloy. *Int J Fatigue*. 2007; **29**(7): 1282-1291. doi: 10.1016/j.ijfatigue.2006.10.003

169. Wang YL, Pan QL, Wei LL *et al.* Effect of retrogression and reaging treatment on the microstructure and fatigue crack growth behavior of 7050 aluminum alloy thick plate. *Mater Des*. 2014; **55**: 857-863. doi: 10.1016/j.matdes.2013.09.063

170. Sharma C, Dwivedi DK, Kumar P. Fatigue behavior of friction stir weld joints of Al–Zn–Mg alloy AA7039 developed using base metal in different temper condition. *Mater Des*. 2014; **64**: 334-344. doi: 10.1016/j.matdes.2014.07.013

171. Baek M-S, Euh K, Lee K-A. Microstructure, tensile and fatigue properties of high strength Al 7075 alloy manufactured via twin-roll strip casting. *J Mater Res Technol*. 2020; **9**(5): 9941-9950. doi: 10.1016/j.jmrt.2020.06.097

172. Bi S, Liu ZY, Xiao BL *et al.* Different fatigue behavior between tension-tension and tension-compression of carbon nanotubes reinforced 7055 Al composite with bimodal structure. *Carbon*. 2021; **184**: 364-374. doi: 10.1016/j.carbon.2021.08.034

173. Liu Z, Zhang H, Yan Z *et al.* Enhanced fatigue performance of aluminum alloy through surface strengthening treatment. *Mater Lett*. 2022; **306**. doi: 10.1016/j.matlet.2021.130864

174. Yang D, Liu Y, Li S *et al.* Effects of aging temperature on microstructure and high cycle fatigue performance of 7075 aluminum alloy. *Journal of Wuhan University of Technology-Mater Sci Ed*. 2017; **32**(3): 677-684. doi: 10.1007/s11595-017-1652-4

175. Yang D, Miao J, Zhang F *et al.* Effects of Prebending Radii on Microstructure and Fatigue Performance of Al-Zn-Mg-Cu Aluminum Alloy after Creep Age Forming. *Metals*. 2019; **9**(6). doi: 10.3390/met9060630

176. Dey S, Das SK, Basumallick A *et al.* The Effect of Pitting on Fatigue Lives of Peak-Aged and Overaged 7075 Aluminum Alloys. *Metall Mater Trans A*. 2010; **41**(13): 3297-3307. doi: 10.1007/s11661-010-0395-z

177. Das P, Jayaganthan R, Chowdhury T *et al.* Fatigue behaviour and crack growth rate of cryorolled Al 7075 alloy. *Mater Sci Eng, A*. 2011; **528**(24): 7124-7132. doi: 10.1016/j.msea.2011.05.021

178. Zheng ZQ, Cai B, Zhai T *et al.* The behavior of fatigue crack initiation and propagation in AA2524-T34 alloy. *Mater Sci Eng, A*. 2011; **528**(4-5): 2017-2022. doi: 10.1016/j.msea.2010.10.085

179. Ni DR, Chen DL, Xiao BL *et al.* Residual stresses and high cycle fatigue properties of friction stir welded SiCp/AA2009 composites. *Int J Fatigue*. 2013; **55**: 64-73. doi: 10.1016/j.ijfatigue.2013.05.010

180. Malarvizhi S, Raghukandan K, Viswanathan N. Fatigue behaviour of post weld heat treated electron beam welded AA2219 aluminium alloy joints. *Mater Des*. 2008; **29**(8): 1562-1567. doi: 10.1016/j.matdes.2007.11.005

181. Nie B, Zhang Z, Zhao Z *et al.* Effect of anodizing treatment on the very high cycle fatigue behavior of 2A12-T4 aluminum alloy. *Mater Des*. 2013; **50**: 1005-1010. doi: 10.1016/j.matdes.2013.03.083

182. Maximov JT, Anchev AP, Duncheva GV *et al.* Impact of slide diamond burnishing additional parameters on fatigue behaviour of 2024-T3 Al alloy. *Fatigue Fract Eng Mater Struct*. 2019; **42**(1): 363-373. doi: 10.1111/ffe.12915

183. Wang C, Chen X, Cheng L *et al.* Very High Cycle Fatigue Properties of 2024 Aluminum Alloy Samples in Three Sizes. *Journal of Physics: Conference Series*. 2021; **2012**(1). doi: 10.1088/1742-6596/2012/1/012032

184. Ludian T, Wagner L. Effect of age-hardening conditions on high-cycle fatigue performance of mechanically surface treated Al 2024. *Mater Sci Eng, A*. 2007; **468-470**: 210-213. doi: 10.1016/j.msea.2006.07.169

185. Fouad Y, Metwally ME. Shot-Peening Effect on High Cycling Fatigue of Al-Cu Alloy. *Metall Mater Trans A*. 2013; **44**(12): 5488-5492. doi: 10.1007/s11661-013-1899-0

186. De PS, Mishra RS, Baumann JA. Characterization of high cycle fatigue behavior of a new generation aluminum lithium alloy. *Acta Mater*. 2011; **59**(15): 5946-5960. doi: 10.1016/j.actamat.2011.06.003

187. Yang C, Zhao Q, Zhang Z *et al.* Nanoparticle additions promote outstanding fracture toughness and fatigue strength in a cast Al–Cu alloy. *Mater Des*. 2020; **186**. doi: 10.1016/j.matdes.2019.108221

188. Patlan V, Vinogradov A, Higashi K *et al.* Overview of fatigue properties of fine grain 5056 Al-Mg alloy processed by equal-channel angular pressing. *Mater Sci Eng A*. 2001; **300**(1): 171-182. doi: 10.1016/S0921-5093(00)01682-8

189. Kikuchi S, Nakamura Y, Nambu K *et al.* Effect of shot peening using ultra-fine particles on fatigue properties of 5056 aluminum alloy under rotating bending. *Mater Sci Eng, A*. 2016; **652**: 279-286. doi: 10.1016/j.msea.2015.11.076

190. Höppel HW, May L, Prell M *et al.* Influence of grain size and precipitation state on the fatigue lives and deformation mechanisms of CP aluminium and AA6082 in the VHCF-regime. *Int J Fatigue*. 2011; **33**(1): 10-18. doi: 10.1016/j.ijfatigue.2010.04.013

191. Zeng L, Shikama T, Takahashi Y *et al.* Fatigue limit of new precipitation-hardened aluminium alloy with distinct fatigue crack propagation limit. *Int J Fatigue*. 2012; **44**: 32-40. doi: 10.1016/j.ijfatigue.2012.06.001

192. Takahashi Y, Shikama T, Nakamichi R *et al.* Effect of additional magnesium on mechanical and high-cycle fatigue properties of 6061-T6 alloy. *Mater Sci Eng, A*. 2015; **641**: 263-273. doi: 10.1016/j.msea.2015.06.051

193. Ochi Y, Masaki K, Matsumura T *et al.* Effects of volume fraction of alumina short fibers on high cycle fatigue properties of Al and Mg alloy composites. *Mater Sci Eng, A*. 2007; **468-470**: 230-236. doi: 10.1016/j.msea.2006.09.122

194. Shikama T, Takahashi Y, Zeng L *et al.* Distinct fatigue crack propagation limit of new precipitation-hardened aluminium alloy. *Scr Mater*. 2012; **67**(1): 49-52. doi: 10.1016/j.scriptamat.2012.03.018

195. Takahashi Y, Shikama T, Yoshihara S *et al.* Study on dominant mechanism of high-cycle fatigue life in 6061-T6 aluminum alloy through microanalyses of microstructurally small cracks. *Acta Mater*. 2012; **60**(6-7): 2554-2567. doi: 10.1016/j.actamat.2012.01.023

196. Sano Y, Masaki K, Gushi T *et al.* Improvement in fatigue performance of friction stir welded A6061-T6 aluminum alloy by laser peening without coating. *Mater Des*. 2012; **36**: 809-814. doi: 10.1016/j.matdes.2011.10.053

197. Zupanič F, Klemenc J, Steinacher M *et al.* Microstructure, mechanical properties and fatigue behaviour of a new high-strength aluminium alloy AA 6086. *J Alloys Compd*. 2023; **941**. doi: 10.1016/j.jallcom.2023.168976

198. Vinogradov A, Washikita A, Kitagawa K *et al.* Fatigue life of fine-grain Al–Mg–Sc alloys produced by equal-channel angular pressing. *Mater Sci Eng, A*. 2003; **349**(1-2): 318-326. doi: 10.1016/s0921-5093(02)00813-4

199. Yi JZ, Gao YX, Lee PD *et al.* Effect of Fe-content on fatigue crack initiation and propagation in a cast aluminum–silicon alloy (A356–T6). *Mater Sci Eng, A*. 2004; **386**(1-2): 396-407. doi: 10.1016/j.msea.2004.07.044

200. Yang B-C, Chen S-F, Song H-W *et al.* Effects of microstructure coarsening and casting pores on the tensile and fatigue properties of cast A356-T6 aluminum alloy: A comparative investigation. *Mater Sci Eng, A*. 2022; **857**. doi: 10.1016/j.msea.2022.144106

201. González R, Martínez DI, González JA *et al.* Experimental investigation for fatigue strength of a cast aluminium alloy. *Int J Fatigue*. 2011; **33**(2): 273-278. doi: 10.1016/j.ijfatigue.2010.09.002

202. Wu Y, Liao H, Tang Y. Enhanced high-cycle fatigue strength of Al–12Si–4Cu-1.2Mn-T6 cast aluminum alloy at room temperature and 350 C. *Mater Sci Eng, A*. 2021; **825**. doi: 10.1016/j.msea.2021.141917

203. Roder O, Wirtz T, Gysler A *et al.* Fatigue properties of Al-Mg alloys with and without scandium. *Mater Sci Eng A*. 1997; **234-236**: 181-184. doi: 10.1016/S0921-5093(97)00224-4

204. Mayer H, Papakyriacou M, Zettl B *et al.* Influence of porosity on the fatigue limit of die cast magnesium and aluminium alloys. *Int J Fatigue*. 2003; **25**(3): 245-256. doi: 10.1016/s0142-1123(02)00054-3

205. Zhu X, Shyam A, Jones J *et al.* Effects of microstructure and temperature on fatigue behavior of E319-T7 cast aluminum alloy in very long life cycles. *Int J Fatigue*. 2006; **28**(11): 1566-1571. doi: 10.1016/j.ijfatigue.2005.04.016

206. Gao YX, Yi JZ, Lee PD *et al.* A micro-cell model of the effect of microstructure and defects on fatigue resistance in cast aluminum alloys. *Acta Mater*. 2004; **52**(19): 5435-5449. doi: 10.1016/j.actamat.2004.07.035

207. Mayer H, Papakyriacou M, Pippan R *et al.* Influence of loading frequency on the high cycle fatigue properties of AlZnMgCu1.5 aluminium alloy. *Mater Sci Eng A*. 2001; **314**(1): 48-54. doi: 10.1016/S0921-5093(00)01913-4

208. Benedetti M, Fontanari V, Santus C *et al.* Notch fatigue behaviour of shot peened high-strength aluminium alloys: Experiments and predictions using a critical distance method. *Int J Fatigue*. 2010; **32**(10): 1600-1611. doi: 10.1016/j.ijfatigue.2010.02.012

209. Verma BB, Atkinson JD, Kumar M. Study of fatigue behaviour of 7475 aluminium alloy. *Bull Mater Sci*. 2001; **24**(2): 231-236. doi: 10.1007/BF02710107

210. De P, Mishra R, Smith C. Effect of microstructure on fatigue life and fracture morphology in an aluminum alloy. *Scr Mater*. 2009; **60**(7): 500-503. doi: 10.1016/j.scriptamat.2008.11.032

211. Gong BS, Zhang ZJ, Qu Z *et al.* Effect of aging state on fatigue property of wrought aluminum alloys. *Int J Fatigue*. 2022; **156**. doi: 10.1016/j.ijfatigue.2021.106682

212. Fintová S, Kuběna I, Trško L *et al.* Fatigue behavior of AW7075 aluminum alloy in ultra-high cycle fatigue region. *Mater Sci Eng, A*. 2020; **774**. doi: 10.1016/j.msea.2020.138922

213. Esmaeili A, Shaeri MH, Noghani MT *et al.* Fatigue behavior of AA7075 aluminium alloy severely deformed by equal channel angular pressing. *J Alloys Compd*. 2018; **757**: 324-332. doi: 10.1016/j.jallcom.2018.05.085

214. de Salvo JGJ, Afonso CRM. Fatigue strength and microstructure evaluation of Al 7050 alloy wires recycled by spray forming, extrusion and rotary swaging. *Trans Nonferrous Met Soc China*. 2020; **30**(12): 3195-3209. doi: 10.1016/s1003-6326(20)65454-1

215. Ma Y, Chen Z, Wang M *et al.* High cycle fatigue behavior of the in-situ TiB2/7050 composite. *Mater Sci Eng, A*. 2015; **640**: 350-356. doi: 10.1016/j.msea.2015.06.023

216. Zhang Q, Zhu Y, Gao X *et al.* Training high-strength aluminum alloys to withstand fatigue. *Nat Commun*. 2020; **11**(1): 5198. doi: 10.1038/s41467-020-19071-7

217. Koike J, Fujiyama N, Ando D *et al.* Roles of deformation twinning and dislocation slip in the fatigue failure mechanism of AZ31 Mg alloys. *Scr Mater*. 2010; **63**(7): 747-750. doi: 10.1016/j.scriptamat.2010.03.021

218. Ishihara S, McEvily AJ, Sato M *et al.* The effect of load ratio on fatigue life and crack propagation behavior of an extruded magnesium alloy. *Int J Fatigue*. 2009; **31**(11-12): 1788-1794. doi: 10.1016/j.ijfatigue.2009.02.034

219. Guo S, Zhou Y, Zhang H *et al.* Thermographic analysis of the fatigue heating process for AZ31B magnesium alloy. *Mater Des*. 2015; **65**: 1172-1180. doi: 10.1016/j.matdes.2014.08.052

220. Sajuri ZB, Miyashita Y, Hosokai Y *et al.* Effects of Mn content and texture on fatigue properties of as-cast and extruded AZ61 magnesium alloys. *Int J Mech Sci*. 2006; **48**(2): 198-209. doi: 10.1016/j.ijmecsci.2005.09.003

221. Khan SA, Miyashita Y, Mutoh Y *et al.* Fatigue behavior of anodized AM60 magnesium alloy under humid environment. *Mater Sci Eng, A*. 2008; **498**(1-2): 377-383. doi: 10.1016/j.msea.2008.08.015

222. Park SH, Hong S-G, Yoon J *et al.* Influence of loading direction on the anisotropic fatigue properties of rolled magnesium alloy. *Int J Fatigue*. 2016; **87**: 210-215. doi: 10.1016/j.ijfatigue.2016.01.026

223. Nascimento L, Yi S, Bohlen J *et al.* High cycle fatigue behaviour of magnesium alloys. *Procedia Engineering*. 2010; **2**(1): 743-750. doi: 10.1016/j.proeng.2010.03.080

224. Tokaji K, Kamakura M, Ishiizumi Y *et al.* Fatigue behaviour and fracture mechanism of a rolled AZ31 magnesium alloy. *Int J Fatigue*. 2004; **26**(11): 1217-1224. doi: 10.1016/j.ijfatigue.2004.03.015

225. Yang F, Yin SM, Li SX *et al.* Crack initiation mechanism of extruded AZ31 magnesium alloy in the very high cycle fatigue regime. *Mater Sci Eng, A*. 2008; **491**(1-2): 131-136. doi: 10.1016/j.msea.2008.02.003

226. Ishihara S, Nan Z, Goshima T. Effect of microstructure on fatigue behavior of AZ31 magnesium alloy. *Mater Sci Eng, A*. 2007; **468-470**: 214-222. doi: 10.1016/j.msea.2006.09.124

227. Uematsu Y, Kakiuchi T, Tamada K *et al.* EBSD analysis of fatigue crack initiation behavior in coarse-grained AZ31 magnesium alloy. *Int J Fatigue*. 2016; **84**: 1-8. doi: 10.1016/j.ijfatigue.2015.11.010

228. Uematsu Y, Tokaji K, Kamakura M *et al.* Effect of extrusion conditions on grain refinement and fatigue behaviour in magnesium alloys. *Mater Sci Eng, A*. 2006; **434**(1-2): 131-140. doi: 10.1016/j.msea.2006.06.117

229. Bhuiyan MS, Mutoh Y, McEvily AJ. The influence of mechanical surface treatments on fatigue behavior of extruded AZ61 magnesium alloy. *Mater Sci Eng, A*. 2012; **549**: 69-75. doi: 10.1016/j.msea.2012.04.007

230. Vaidya AR, Lewandowski JJ. Effects of SiCp size and volume fraction on the high cycle fatigue behavior of AZ91D magnesium alloy composites. *Mater Sci Eng A*. 1996; **220**(1): 85-92. doi: 10.1016/S0921-5093(96)10464-0

231. Ni DR, Wang D, Feng AH *et al.* Enhancing the high-cycle fatigue strength of Mg–9Al–1Zn casting by friction stir processing. *Scr Mater*. 2009; **61**(6): 568-571. doi: 10.1016/j.scriptamat.2009.05.023

232. Mayer H, Papakyriacou M, Zettl B *et al.* Endurance limit and threshold stress intensity of die cast magnesium and aluminium alloys at elevated temperatures. *Int J Fatigue*. 2005; **27**(9): 1076-1088. doi: 10.1016/j.ijfatigue.2005.02.002

233. Ishihara S, Namito T, Yoshifuji S *et al.* On fatigue lives of diecast and extruded Mg alloys. *Int J Fatigue*. 2012; **35**(1): 56-62. doi: 10.1016/j.ijfatigue.2010.11.023

234. Lv F, Yang F, Duan QQ *et al.* Fatigue properties of rolled magnesium alloy (AZ31) sheet: Influence of specimen orientation. *Int J Fatigue*. 2011; **33**(5): 672-682. doi: 10.1016/j.ijfatigue.2010.10.013

235. Liu W, Jiang L, Cao L *et al.* Fatigue behavior and plane-strain fracture toughness of sand-cast Mg–10Gd–3Y–0.5Zr magnesium alloy. *Mater Des*. 2014; **59**: 466-474. doi: 10.1016/j.matdes.2014.03.026

236. Yu D, Zhang D, Sun J *et al.* High cycle fatigue behavior of extruded and double-aged Mg-6Zn-1Mn alloy. *Mater Sci Eng, A*. 2016; **662**: 1-8. doi: 10.1016/j.msea.2016.02.079

237. He Z, Fu P, Wu Y *et al.* High cycle fatigue behavior of as-cast Mg96.34Gd2.5Zn1Zr0.16 alloy fabricated by semi-continuous casting. *Mater Sci Eng, A*. 2013; **587**: 72-78. doi: 10.1016/j.msea.2013.08.046

238. Li ZM, Wang QG, Luo AA *et al.* Improved high cycle fatigue properties of a new magnesium alloy. *Mater Sci Eng, A*. 2013; **582**: 170-177. doi: 10.1016/j.msea.2013.06.001

239. Li ZM, Fu PH, Peng LM *et al.* Comparison of high cycle fatigue behaviors of Mg–3Nd–0.2Zn–Zr alloy prepared by different casting processes. *Mater Sci Eng, A*. 2013; **579**: 170-179. doi: 10.1016/j.msea.2013.05.040

240. Liu WC, Dong J, Zhang P *et al.* Improvement of fatigue properties by shot peening for Mg–10Gd–3Y alloys under different conditions. *Mater Sci Eng, A*. 2011; **528**(18): 5935-5944. doi: 10.1016/j.msea.2011.04.004

241. Li Z, Wang Q, Luo AA *et al.* Fatigue strength dependence on the ultimate tensile strength and hardness in magnesium alloys. *Int J Fatigue*. 2015; **80**: 468-476. doi: 10.1016/j.ijfatigue.2015.07.001

242. Yang F, Lv F, Yang XM *et al.* Enhanced very high cycle fatigue performance of extruded Mg–12Gd–3Y–0.5Zr magnesium alloy. *Mater Sci Eng, A*. 2011; **528**(6): 2231-2238. doi: 10.1016/j.msea.2010.12.092

243. Li Z, Fu P, Peng L *et al.* Influence of solution temperature on fatigue behavior of AM-SC1 cast magnesium alloy. *Mater Sci Eng, A*. 2013; **565**: 250-257. doi: 10.1016/j.msea.2012.12.035

244. Li Z, Wang Q, Luo AA *et al.* High Cycle Fatigue of Cast Mg-3Nd-0.2Zn Magnesium Alloys. *Metall Mater Trans A*. 2013; **44**(11): 5202-5215. doi: 10.1007/s11661-013-1843-3

245. Dong J, Liu WC, Song X *et al.* Influence of heat treatment on fatigue behaviour of high-strength Mg–10Gd–3Y alloy. *Mater Sci Eng, A*. 2010; **527**(21-22): 6053-6063. doi: 10.1016/j.msea.2010.06.030

246. Xu DK, Liu L, Xu YB *et al.* The crack initiation mechanism of the forged Mg–Zn–Y–Zr alloy in the super-long fatigue life regime. *Scr Mater*. 2007; **56**(1): 1-4. doi: 10.1016/j.scriptamat.2006.09.006

247. Eifert AJ, Thomas JP, Rateick RG. Influence of anodization on the fatigue life of WE43A-T6 magnesium. *Scr Mater*. 1999; **40**(8): 929-935. doi: 10.1016/S1359-6462(99)00040-8

248. Wang SD, Xu DK, Wang BJ *et al.* Effect of solution treatment on the fatigue behavior of an as-forged Mg-Zn-Y-Zr alloy. *Sci Rep*. 2016; **6**: 23955. doi: 10.1038/srep23955

249. Pan QS, Lu QH, Lu L. Fatigue behavior of columnar-grained Cu with preferentially oriented nanoscale twins. *Acta Mater*. 2013; **61**(4): 1383-1393. doi: 10.1016/j.actamat.2012.11.015

250. Freudenberger J, Klauß HJ, Heinze K *et al.* Fatigue of highly strengthened Cu–Ag alloys. *Int J Fatigue*. 2008; **30**(3): 437-443. doi: 10.1016/j.ijfatigue.2007.04.009

251. Lukáš P, Kunz L, Navrátilová L *et al.* Fatigue damage of ultrafine-grain copper in very-high cycle fatigue region. *Mater Sci Eng, A*. 2011; **528**(22-23): 7036-7040. doi: 10.1016/j.msea.2011.06.001

252. Khatibi G, Horky J, Weiss B *et al.* High cycle fatigue behaviour of copper deformed by high pressure torsion. *Int J Fatigue*. 2010; **32**(2): 269-278. doi: 10.1016/j.ijfatigue.2009.06.017

253. Kunz L, Lukáš P, Navrátilová L. Strain localization and fatigue crack initiation in ultrafine-grained copper in high- and giga-cycle region. *Int J Fatigue*. 2014; **58**: 202-208. doi: 10.1016/j.ijfatigue.2013.04.027

254. Phung NL, Favier V, Ranc N *et al.* Very high cycle fatigue of copper: Evolution, morphology and locations of surface slip markings. *Int J Fatigue*. 2014; **63**: 68-77. doi: 10.1016/j.ijfatigue.2014.01.007

255. Liu R, Tian YZ, Zhang ZJ *et al.* Exploring the fatigue strength improvement of Cu-Al alloys. *Acta Mater*. 2018; **144**: 613-626. doi: 10.1016/j.actamat.2017.11.019

256. Zhang ZJ, Pang JC, Zhang ZF. Optimizing the fatigue strength of ultrafine-grained Cu-Zn alloys. *Mater Sci Eng, A*. 2016; **666**: 305-313. doi: 10.1016/j.msea.2016.04.076

257. Pang JC, Duan QQ, Wu SD *et al.* Fatigue strengths of Cu–Be alloy with high tensile strengths. *Scr Mater*. 2010; **63**(11): 1085-1088. doi: 10.1016/j.scriptamat.2010.08.009

258. Zhaokuang C, Jinjiang Y, Xiaofeng S *et al.* High cycle fatigue behavior of a directionally solidified Ni-base superalloy DZ951. *Mater Sci Eng, A*. 2008; **496**(1-2): 355-361. doi: 10.1016/j.msea.2008.05.031

259. Chen Y, Kong W, Yuan C *et al.* The effects of temperature and stress on the high-cycle fatigue properties of a Ni-based wrought superalloy. *Int J Fatigue*. 2023; **172**. doi: 10.1016/j.ijfatigue.2023.107669

260. Tao X, Tan K, Liang J *et al.* Pt-Al bond coat dependence on the high-cycle fatigue rupture and deformation mechanisms of a fourth-generation single crystal superalloy at various temperatures. *Mater Des*. 2023; **229**. doi: 10.1016/j.matdes.2023.111880

261. Li X, Li W, Hu T *et al.* A novel very-high-cycle-fatigue life prediction model with interior microstructure induced cracking behavior of Inconel-713C superalloy at 25 °C, 750 °C and 1000 °C. *Theor Appl Fract Mech*. 2023; **123**. doi: 10.1016/j.tafmec.2022.103705

262. Yu C, Huang Z, Zhang Z *et al.* Effects of sandblasting and HIP on very high cycle fatigue performance of SLM-fabricated IN718 superalloy. *J Mater Res Technol*. 2022; **18**: 29-43. doi: 10.1016/j.jmrt.2022.02.077

263. Wan H-Y, Zhou Z-J, Li C-P *et al.* Enhancing Fatigue Strength of Selective Laser Melting-Fabricated Inconel 718 by Tailoring Heat Treatment Route. *Adv Eng Mater*. 2018; **20**(10). doi: 10.1002/adem.201800307

264. Kobayashi K, Yamaguchi K, Hayakawa M *et al.* High-temperature fatigue properties of austenitic superalloys 718, A286 and 304L. *Int J Fatigue*. 2008; **30**(10-11): 1978-1984. doi: 10.1016/j.ijfatigue.2008.01.004

265. Zimmermann M, Stöcker C, Christ HJ. On the effects of particle strengthening and temperature on the VHCF behavior at high frequency. *Int J Fatigue*. 2011; **33**(1): 42-48. doi: 10.1016/j.ijfatigue.2010.05.007

266. Qin Z, Li B, Chen R *et al.* Effect of shot peening on high cycle and very high cycle fatigue properties of Ni-based superalloys. *Int J Fatigue*. 2023; **168**. doi: 10.1016/j.ijfatigue.2022.107429

267. Kawagoishi, Chen, Nisitani. Fatigue strength of Inconel 718 at elevated temperatures. *Fatigue Fract Eng Mater Struct*. 2000; **23**(3): 209-216. doi: 10.1046/j.1460-2695.2000.00263.x

268. Yu X, Lin X, Wang Z *et al.* Room and high temperature high-cycle fatigue properties of Inconel 718 superalloy prepared using laser directed energy deposition. *Mater Sci Eng, A*. 2021; **825**. doi: 10.1016/j.msea.2021.141865

269. Wan A, Xiong J, Lyu Z *et al.* High-cycle fatigue behavior of Co-based superalloy 9CrCo at elevated temperatures. *Chin J Aeronaut*. 2016; **29**(5): 1405-1413. doi: 10.1016/j.cja.2016.01.009

270. Ghomsheh MZ, Khatibi G, Weiss B *et al.* High cycle fatigue deformation mechanisms of a single phase CrMnFeCoNi high entropy alloy. *Mater Sci Eng, A*. 2020; **777**. doi: 10.1016/j.msea.2020.139034

271. Kim Y-K, Ham G-S, Kim HS *et al.* High-cycle fatigue and tensile deformation behaviors of coarse-grained equiatomic CoCrFeMnNi high entropy alloy and unexpected hardening behavior during cyclic loading. *Intermetallics*. 2019; **111**. doi: 10.1016/j.intermet.2019.106486

272. Suzuki K, Koyama M, Hamada S *et al.* Planar slip-driven fatigue crack initiation and propagation in an equiatomic CrMnFeCoNi high-entropy alloy. *Int J Fatigue*. 2020; **133**. doi: 10.1016/j.ijfatigue.2019.105418

273. Liu K, Nene SS, Frank M *et al.* Metastability-assisted fatigue behavior in a friction stir processed dual-phase high entropy alloy. *Mater Res Lett*. 2018; **6**(11): 613-619. doi: 10.1080/21663831.2018.1523240

274. Liu K, Nene SS, Frank M *et al.* Extremely high fatigue resistance in an ultrafine grained high entropy alloy. *Appl Mat Today*. 2019; **15**: 525-530. doi: 10.1016/j.apmt.2019.04.001

275. Shukla S, Wang T, Cotton S *et al.* Hierarchical microstructure for improved fatigue properties in a eutectic high entropy alloy. *Scr Mater*. 2018; **156**: 105-109. doi: 10.1016/j.scriptamat.2018.07.022

276. Liu K, Komarasamy M, Gwalani B *et al.* Fatigue behavior of ultrafine grained triplex Al0.3CoCrFeNi high entropy alloy. *Scr Mater*. 2019; **158**: 116-120. doi: 10.1016/j.scriptamat.2018.08.048

277. Tang Z, Yuan T, Tsai C-W *et al.* Fatigue behavior of a wrought Al0.5CoCrCuFeNi two-phase high-entropy alloy. *Acta Mater*. 2015; **99**: 247-258. doi: 10.1016/j.actamat.2015.07.004

278. Tian YZ, Sun SJ, Lin HR *et al.* Fatigue behavior of CoCrFeMnNi high-entropy alloy under fully reversed cyclic deformation. *J Mater Sci Technol*. 2019; **35**(3): 334-340. doi: 10.1016/j.jmst.2018.09.068
